# Oxandrastins: Antibacterial Meroterpenes from an Australian Mud Dauber Wasp Nest-Associated Fungus, *Penicillium* sp. CMB-MD14

**DOI:** 10.3390/molecules26237144

**Published:** 2021-11-25

**Authors:** Ahmed H. Elbanna, Zeinab G. Khalil, Robert J. Capon

**Affiliations:** 1 Institute for Molecular Bioscience, The University of Queensland, Brisbane, QLD 4072, Australia; ahmed.elbanna@pharma.cu.edu.eg (A.H.E.); z.khalil@uq.edu.au (Z.G.K.); 2 Current address: Department of Pharmacognosy, Faculty of Pharmacy, Cairo University, Cairo 11562, Egypt

**Keywords:** wasp nest-associated fungi, *Penicillium* sp. CMB-MD14, antibacterial, vancomycin-resistant enterococci, bioassay guided investigation, meroterpenoids, oxandrastins, austalides

## Abstract

The ethyl acetate extract of an ISP-2 agar cultivation of the wasp nest-associated fungus *Penicillium* sp. CMB-MD14 exhibited promising antibacterial activity against vancomycin-resistant enterococci (VRE), with a bioassay guided chemical investigation yielding the new meroterpene, oxandrastin A (**1**), the first andrastin-*like* metabolite with an extra oxygenation at C-2. A culture media optimisation strategy informed a scaled-up rice cultivation that yielded **1**, together with three new oxandrastins B–D (**2**–**4**), two known andrastins C (**5**) and F (**6**), and a new meroterpene of the austalide family, isoaustalide F (**7**). Structures of **1**–**7** were assigned based on detailed spectroscopic analysis and chemical interconversion. A GNPS molecular networking analysis of the rice cultivation extract detected the known austalides B (**8**), H (**9**), and H acid (**10**), tentatively identified based on molecular formulae and co-clustering with **7**. That the anti-VRE properties of the CMB-MD14 extract were exclusively attributed to **1** (IC_50_ 6.0 µM, MIC_99_ 13.9 µM), highlights the importance of the 2-OAc and 3-OAc moieties to the oxandrastin anti-VRE pharmacophore.

## 1. Introduction

The widespread emergence of antibiotic resistance is seriously undermining the capacity of modern antibiotics to protect against infectious disease. Antibiotic resistance is not just a problem for patients presenting with a primary infection, as secondary infections also drive high levels of morbidity and mortality. For example, patients with genetic, cardiovascular, and/or respiratory diseases, or with compromised immune systems, or even post-operative patients, are at risk from secondary infection. The magnitude of the antibiotic crisis is further exacerbated by the fact that most pharmaceutical companies have exited the *antibiotic space*, leaving an under resourced and depleted drug development pipeline desperately in need of innovation and renewal. As a result, current infection control is hostage to the residual efficacy of a handful of resistance compromised antibiotics.

For the latter half of the last century, science and industry successfully exploited microbial chemical defences, fuelling a knowledge revolution that inspired new pharmaceuticals and agrochemicals, driving global commerce, and improving quality of life. This led to some of the most widely recognised and successful antibiotic classes, including the penicillins, erythromycins, cephalosporins, tetracyclines, streptomycins, vancomycins, and rifampicins. Notwithstanding this success, when confronted by lower returns on investment and the perception of a near exhausted microbial resource, late last century industry turned elsewhere for inspiration. Two decades on, and confronted by surging antibiotic resistance, and a community demand for better, safer, and more effective antibiotics, the need for inspiration is more urgent than ever.

**Figure 1:**
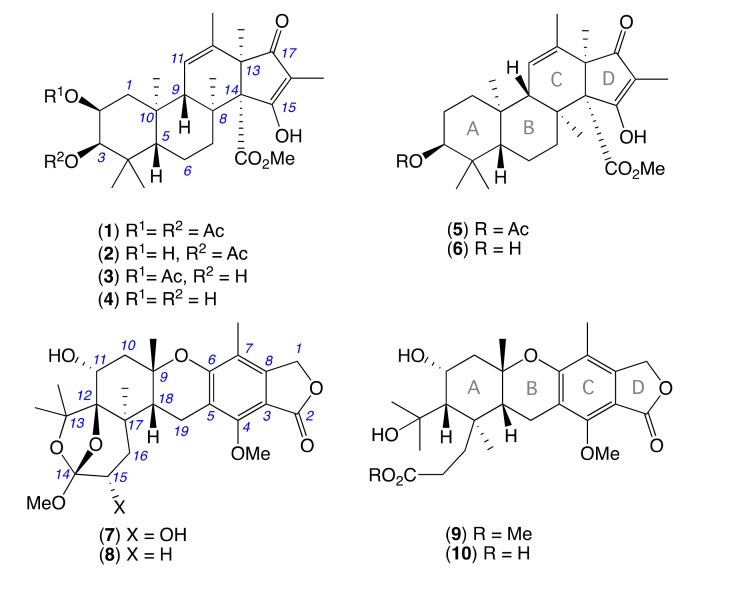
Metabolites **1**–**10** from *Penicillium* sp. CMB-MD14.

As part of our ongoing investigations into the chemistry of Australian fungi, we assembled a library of fungi co-isolated from a mud dauber wasp (×15), and a mud dauber wasp nest (×21), both sourced from an urban location in Pullenvale, Queensland, Australia. Collectively, this wasp-derived fungal library proved to be an excellent source of novel natural products with *Aspergillus* sp. CMB-W031, yielding an unprecedented nitro depsi-peptide diketopiperazine (waspergillamide A) [1]. *Talaromyces* sp. CMB-W045 yielding new non-cytotoxic siderophores (talarazines A–E) [2], a new a *p*-terphenyl (talarophenol sulfate), and polyketides (talarophilones) [3]. *Penicillium* sp. CMB-MD22 yielded an extensive family of stereo-complex and selectively antifungal neobulgarone bianthrones [4]. Turning our attention to the need for new antibiotic classes, and prompted by promising antibacterial activity against vancomycin-resistant enterococci (VRE), this current report describes an investigation into the antibacterial natural products of the mud dauber wasp nest-derived *Penicillium* sp. CMB-MD14. Bioassay guided fractionation of a scaled-up ISP-2 agar cultivation yielded the new anti-VRE meroterpene **1**, closely related to the known antibacterial inactive fungal meroterpene andrastins [5,6] as well as the atlantinones [7,8], citreohybridones, isocitreohybridones and citreohybriddiones [9,10]. The first andrastin-*like* metabolite to be reported with C-2 oxygenation, **1** was assigned the trivial name oxandrastin A. To better explore the anti-VRE oxandrastin pharmacophore a media optimisation strategy was employed, with a scaled-up rice cultivation of CMB-MD14 yielding oxandrastin A (**1**), as well as three new oxandrastins B–D (**2–4**), two known andrastins C (**5**), F (**6**) [5,6] and a new meroterpene of the austalide family, isoaustalide F (**7**) (Figure 1).

**Figure 2:**
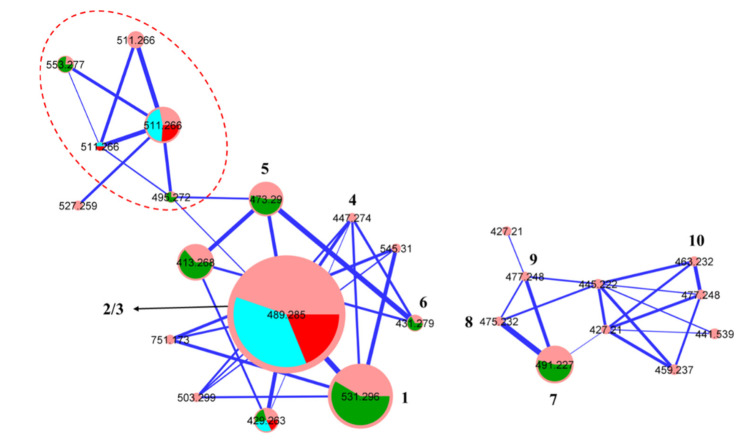
GNPS molecular networking revealing cluster for (left) oxandrastins/adrastins and (right) austalides; (pink) CMB-MD14 rice extract; (green) **1** and **5**–**7**; (blue) **2**; (red) **3**; (red dashed) sodiated adducts of **1**–**3** and **5**–**6**.

A GNPS molecular networking analysis of the unfractionated CMB-MD14 rice cultivation extract confirmed separate clusters for oxandrastins/andrastins and isoaustalides/austalides (Figure 2), with the latter including nodes *tentatively* attributed to the known austalides B (**8**) [11,12], H (**9**) [13] and H acid (**10**) [14]. Subsequent UPLC-MS analysis with single ion extraction (SIE) detected **1**–**10** in the fresh extract, which together with GNPS analyses confirmed their natural product status. Structures were assigned **1**–**7** based on detailed spectroscopic analysis and chemical interconversion, and *tentatively* to **8**–**10** on the basis of molecular formulae and GNPS co-clustering with **7**. An account of these structure determinations together with commentary on anti-VRE structure activity and biosynthetic relationships is outlined below.

## 2. Results and Discussion

**Figure 3:**
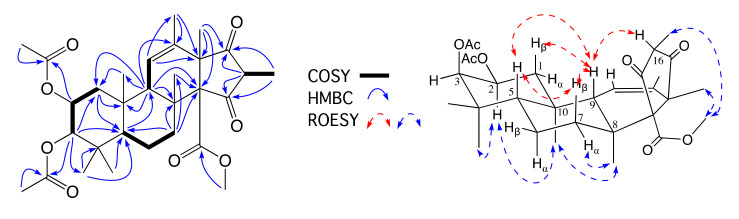
Diagnostic 2D NMR (CDCl_3_) correlations for oxandrastin A (**1**).

**Figure 4:**
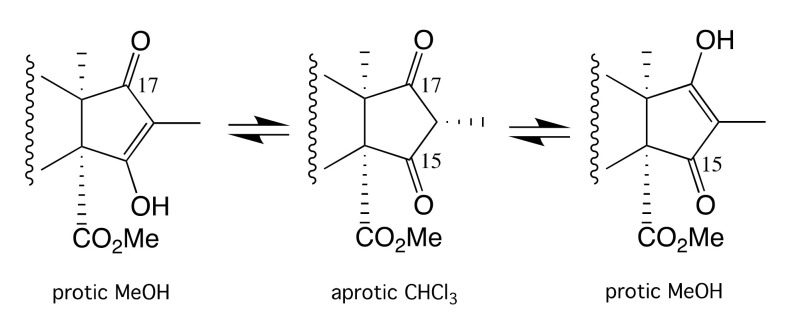
Tautomeric keto-enol forms of **1** present in aprotic versus protic solvents.

HRESI(+)MS analysis of **1** revealed a molecular formula (C_30_H_42_O_8_) requiring 10 double bond equivalents (DBE). The 1D NMR (CDCl_3_) data for **1** (Table S1, Figures S7 and S8) revealed three ester (δ_C_ 170.6, 2-CO; 171.0, 3-CO, and 168.4, 14-CO_2_) and two ketone (δ_C_ 210.7, C-15, and δ_C_ 209.5, C-17) carbonyls, and a trisubstituted double bond (δ_H_ 5.58, br s, H-11; δ_C_ 126.8, C-11; δ_C_ 135.1, C-12), accounting for 6 DBE and requiring that **1** be tetracyclic. Diagnostic 2D NMR COSY and HMBC correlations established the planar structure for **1**, while ROESY correlations defined the full relative configuration (Figure 3). More specifically, ROESY correlations between H-2, H_3α_-4, H_3_-10, and H_3_-8 confirmed a co-facial (α) orientation, while correlations between H_β_-1, H-5, H_β_-7, H-9, and H-16 confirmed a co-facial (β) orientation. Supportive of these assignments, the magnitude of *J*_1α,2_ (4.0 Hz), *J*_1β,2_ (12.4 Hz), and *J*_2,3_ (3.1 Hz) confirmed the axial and equatorial orientation of H-2 and H-3, respectively, and the structure for oxandrastin A (**1**) (relative configuration only) as shown. Of note, the NMR (methanol-*d*_4_) data for **1** (Table 1, Table 2 and Table S2; Figures S10 and S11) revealed an alternate ring D enol tautomer and, as the ^13^C NMR (MeOH-*d*_4_) resonances for the C-15 and C-17 were not detectable (broadened), it seemed likely that ring D was an equilibrating mixture of tautomers (Figure 4). A search of the literature revealed that many of the above mentioned related fungal meroterpenes (e.g., andrastins and citreohybridonol) exhibited a comparable solvent dependent ring D keto-enol tautomerism [5,7,10,15]. Significantly, this solvent effect was of added importance when acquiring optical measurements. For example, the [α]_D_ for **1** measured in CHCl_3_ (–64) was larger than that measured in MeOH (–18.3).

**Figure 5:**
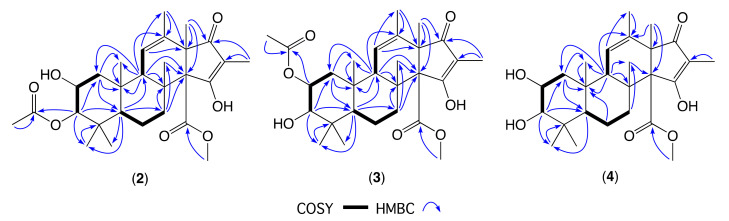
Diagnostic 2D NMR (methanol-*d*_4_) correlations for oxandrastins B–D (**2**–**4**).

HRMS(+)ESI analysis of **2** and **3** revealed isomeric molecular formulae (C_28_H_40_O_7_) suggestive of deacetylated analogues of **1**. As **2** exhibited limited solubility in CDCl_3_, comparison of the 1D NMR (methanol-*d*_4_) data for **2** (Table 1, Table 2 and Table S3; Figures S13 and S14) with **1** confirmed the loss of resonances for an acetyl moiety, with a corresponding upfield shift of H-2 in **2** (Δδ_H_ = −1.18) attributed to conversion of the 2-OAc in **1** to a 2-OH in **2**. In addition, values for *J*_1α,2_ (4.1 Hz), *J*_1β,2_ (12.1 Hz), and *J*_2,3_ (2.8 Hz) in **2** confirmed an axial H-2 and equatorial H-3 in common with **1**. Diagnostic 2D NMR COSY and HMBC correlations (Figure 5) supported the planar structure, while ROESY correlations permitted assignment of the complete relative configuration for oxandrastin B (**2**) as shown.

Comparison of the NMR (methanol-*d*_4_) data for **3** (Table 1, Table 2 and Table S4; Figures S16 and S17) with **1** also confirmed the loss of resonances for an acetyl moiety, with a corresponding upfield shift of H-3 in **3** (Δδ_H_ = −1.52) attributed to conversion of the 3-OAc in **1** to a 3-OH in **3**. In addition, values for *J*_1α,2_ (4.1 Hz), *J*_1β,2_ (12.1 Hz), and *J*_2,3_ (2.7 Hz) in **3** confirmed an axial H-2 and equatorial H-3 in common with **1**. Diagnostic 2D NMR COSY and HMBC correlations (Figure 5) supported the planar structure, while ROESY correlations permitted assignment of the complete relative configuration for oxandrastin C (**3**) as shown.

A global natural products social (GNPS) molecular networking analysis of the unfractionated rice cultivation extract detected an array of minor metabolites including **4** (Figure 2 and Figure S34), speculated on the basis of its molecular formula (C_26_H_38_O_6_) to be the deacetylated homologue of the monoacetates **2** and **3**. Although only an exceptionally minor metabolite in the extract, alkaline hydrolysis of **2,** yielded pure semi-synthetic **4,** which co-eluted on UPLC and had an MS/MS fragmentation in common with the natural product **4** (Figures S4 and S5), which was identified by detailed spectroscopic analysis. Comparison of the NMR (methanol-*d*_4_) data for **4** (Table 1, Table 2 and Table S5; Figures S19–S21) with **1** confirmed the loss of two acetyl moieties, with corresponding upfield shifts of H-2 and H-3 in **4** (Δδ_H_ = −1.31 and −1.64, respectively) attributed to conversion of the 2-OAc and 3-OAc in **1** to 2-OH and 3-OH moieties in **4**. Diagnostic 2D NMR COSY and HMBC correlations (Figure 5), together with chemical interconversion with **2,** permitted assignment of the structure for oxandrastin D (**4**) as shown (relative configuration).

**Figure 6:**
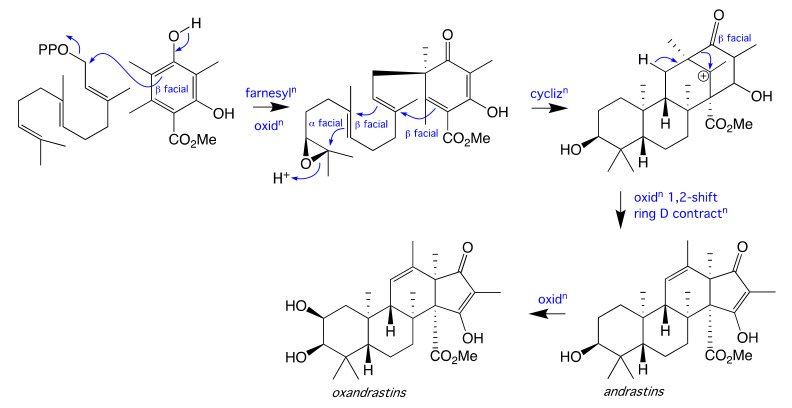
Proposed biosynthesis of the meroterpene andrastins and oxandrastins.

Spectroscopic analysis of the co-metabolites **5** (C_28_H_40_O_6_) and **6** (C_26_H_38_O_5_) (Tables S6 and S7; Figures S23, S24, S26 and S27) readily identified them as the known fungal metabolites andrastin C and andrastin F [5,6] respectively. Significantly, as the absolute configuration of **5** had been established in 1996 by X-ray analysis of its co-metabolite andrastin A [5] and the optical properties of our re-isolation ([α]_D_ = −16.5 in MeOH; −134.4 in CHCl_3_) corresponded well with those reported in the scientific literature ([α]_D_ = −30.3 in MeOH; −143.4 in CHCl_3_), on biosynthetic grounds we inferred the absolute configuration of oxandrastins A–D (**1**–**4**), as shown. A plausible biosynthesis of the meroterpene andrastins and oxandrastins was summarised in Figure 6.

**Figure 7:**
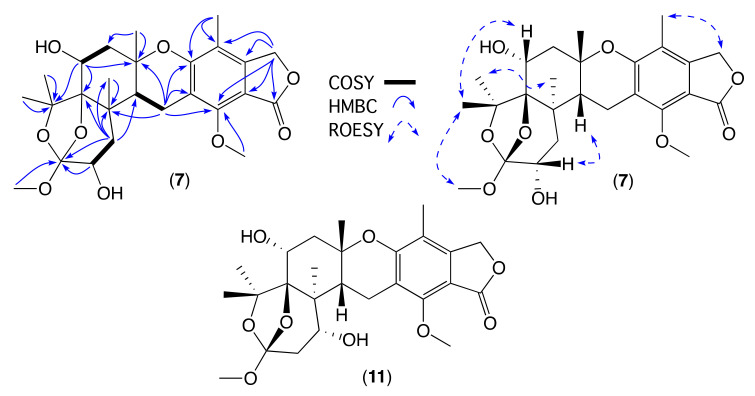
Diagnostic 2D NMR (methanol-*d*_4_) correlations for isoaustalide F (**7**), and the structure for austalide F (**11**).

The GNPS analysis of CMB-MD14 (Figure 2 and Figure S35) disclosed a second family of meroterpenes, which following fractionation, yielded **7** (C_26_H_34_O_9_) isomeric and sharing a common UV-vis chromophore with the known fungal metabolite austalide F (**11**) (Figure 7). Comparison of the 1D NMR (methanol-*d*_4_) data for **7** (Table 3 and Table S8; Figures S29 and S30) with **11** suggested the presence of a C-15 2°-OH in **7** (H-15 δ_H_ 3.71, dd, 10.5, and 5.9; C-15 δ_C_ 68.8) as opposed to the C-16 2°-OH in **11**. This hypothesis was confirmed by 2D NMR correlations, which together with diagnostic ROESY correlations established the structure and relative configuration (Figure 7), with the absolute configuration for isoaustalide F (**7**) assigned on the basis of comparable experimental ECD spectra and specific rotations ([α]_D_ = −43), with those reported for the known austalides F ([α]_D_ = −57.7) [11]. A ([α]_D_ = −84.4) [11] and T ([α]_D_ = −88) [16] (Figure S32). The absolute configurations of known austalides have been confirmed by either ECD calculations, an X-ray structure analysis for a derivatized austalide D, or total synthesis of some analogues, such as austalide B [14,16,17,18].

**Figure 8:**
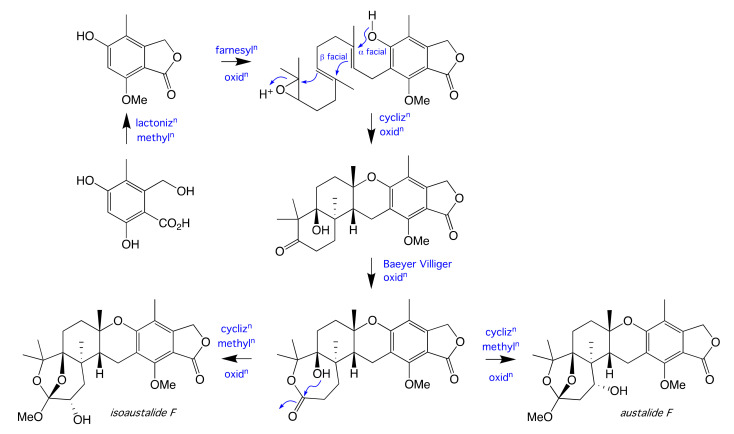
Proposed biosynthesis of the meroterpene austalide F and isoaustalide F (**7**).

Minor GNPS nodes within the CMB-MD14 austalide family (Figure 2 and Figures S35) were tentatively identified based on correlations within the GNPS databases as the known austalides B (**8**) (*m/z* 475), H (**9**) (*m/z* 477) and H acid (**10**) (*m/z* 463). Both **8** and **9** were firstly reported from the toxigenic *Aspergillus ustus* (Bainier) Thom. and Church (strain MRC 1163) [11,12,13], while **10** was later isolated from the alga-derived fungi *Penicillium thomii* (Maire) and *Penicillium lividum* (Westling) [14]. The natural product status of **8**–**10** in the CMB-MD14 extract was confirmed by UPLC-QTOF analysis with single ion extraction (SIE) monitoring (Figure S36). A plausible biosynthesis linking austalide F (**11**) and isoaustalide F (**7**) is summarised in Figure 8.

The CMB-MD14 metabolites **1**–**7** did not exhibit growth inhibitory activity (IC_50_ > 30 μM) against the Gram −ve bacteria *Escherichia coli* ATCC11775, the Gram +ve bacteria *Staphylococcus aureus* ATCC25923, the fungus *Candida albicans* ATCC10231, or human colorectal (SW620) and lung (NCI-H460) carcinoma cells (Figures S37 and S38). Significantly, the anti-VRE properties of the CMB-MD14 extract were exclusively attributed to **1**, which exhibited growth inhibitory activity against a clinical isolate of vancomycin resistant *Enterococcus* (VRE) (IC_50_ 6.0 µM, MIC_99_ 13.9 µM), with slightly improved activity against a vancomycin susceptible *Enterococcus faecalis* ACM 5184 (IC_50_ 1.9 µM, MIC_99_ 6.1 µM) (Figure S37), suggesting that 2-OAc and 3-OAc moieties were pivotal to the anti-VRE properties of the oxandrastin pharmacophore.

## 3. Materials and Methods

### 3.1. General Experimental Procedures

Chiroptical measurements ([α]_D_) were obtained on a JASCO P-1010 polarimeter (JASCO International Co. Ltd., Tokyo, Japan) in a 100 × 2 mm cell at specified temperatures. Electronic Circular Dichroism (ECD) measurement were obtained on a JASCO J-810 spectropolarimeter (JASCO International Co. Ltd., Tokyo, Japan) in a 0.1 cm path-length cell. Nuclear magnetic resonance (NMR) spectra were acquired on a Bruker Avance 600 MHz spectrometer (Bruker Pty. Ltd., Alexandria, NSW, Australia) with either a 5 mm PASEL ^1^H/D-^13^C Z-Gradient probe or 5 mm CPTCI ^1^H/^19^F-^13^C/^15^N/DZ-Gradient cryoprobe. In all cases, spectra were acquired at 25 °C deuterated solvents as indicated, with referencing to residual ^1^H or ^13^C signals. High-resolution ESIMS spectra were obtained on a Bruker micrOTOF mass spectrometer (Bruker Daltonik Pty. Ltd., Preston, VIC, Australia) by direct injection in MeOH at 3 μL/min using sodium formate clusters as an internal calibrant. Liquid chromatography-diode array-mass spectrometry (HPLC-DAD-MS) data were acquired either on an Agilent 1260 series separation module equipped with an Agilent G6125B series LC/MSD mass detector (Agilent Technologies Inc., Mulgrave, VIC, Australia) and diode array detector or on Shimadzu LCMS-2020 LCMS. Semi-preparative HPLCs were performed using Agilent 1100 series HPLC instruments (Agilent Technologies Inc., Mulgrave, VIC, Australia) with corresponding detectors, fraction collectors, and software inclusively. UPLC chromatograms were obtained on Agilent 1290 infinity UPLC system equipped with diode array multiple wavelength detector (Zorbax C_8_ RRHD 1.8 μm, 50 × 2.1 mm column, 0.417 mL/min with a 2.50 min gradient from 90% H_2_O/MeCN to MeCN with a constant 0.01% TFA modifier). UPLC-QTOF analysis was performed on UPLC-QTOF instrument comprising an Agilent 1290 Infinity II UPLC (Zorbax C_8_ RRHD 1.8 μm, 50 × 2.1 mm column, eluting at 0.417 mL/min with a 2.50 min gradient elution from 90% H_2_O/MeCN to 100% MeCN with a constant 0.1% formic acid modifier) coupled to an Agilent 6545 Q-TOF. MS/MS analysis was performed on the same instrument for ions detected in the full scan, at an intensity above 1000 counts at 10 scans/s, with an isolation width of 4 ~m/z using a fixed collision energy and a maximum of 3 selected precursors per cycle. Chemicals were purchased from Sigma-Aldrich or Merck unless otherwise specified. Analytical-grade solvents were used for solvent extractions. Chromatography solvents were of HPLC grade supplied by Labscan or Sigma-Aldrich and filtered/degassed through 0.45 μm polytetrafluoroethylene (PTFE) membrane prior to use. Deuterated solvents were purchased from Cambridge Isotopes. Microorganisms were manipulated under sterile conditions using a Laftech class II biological safety cabinet and incubated in either MMM Friocell incubators (Lomb Scientific, NSW, Australia) or an Innova 42R incubator shaker (John Morris, NSW, Australia).

### 3.2. Fungal Isolation

The fungus *Penicillium sp.* CMB-MD14 was isolated from a specimen of mud dauber wasp nests collected from an urban location in Pullenvale, Queensland, Australia, by cultivation on ISP-2 agar plate at 26.5 °C for 8 days.

### 3.3. Fungal Taxonomy

Genomic DNA for CMB-MD14 was extracted from its mycelia using the DNeasy Plant Mini Kit (Qiagen), as per the manufacturers protocol. The 18s rRNA gene was amplified by PCR using the universal primers ITS-1 (5ʹ-TCCGTAGGTGAACCTGCGG-3ʹ) and ITS-4 (5ʹ-TCCTCCGCTTATTGATATGC-3ʹ) purchased from Sigma-Aldrich. The PCR mixture (50 μL) contained genomic DNA (2 μL, 20–40 ng), EmeraldAmpn GT PCR Master Mix (2X Premix) (25 μL), primer (0.2 μM, each), and H_2_O (up to 50 μL). PCR was performed using the following conditions: initial denaturation at 95 °C for 2 min, 30 cycles in series of 95 °C for 20 s (denaturation), 56 °C for 20 s (annealing), and 72 °C for 30 s (extension), followed by one cycle at 72 °C for 5 min. PCR products were purified with PCR purification kit (Qiagen, Victoria, Australia). Amplification products were examined by agarose gel electrophoresis. The DNA sequencing was performed by the Australian Genome Research Facility (AGRF) at The University of Queensland. A BLAST analysis (NCBI database) on the resulting ITS gene sequence (GenBank accession no. MW185842) for CMB-MD14 revealed 99% identity with the fungal strain *Penicillium panissanguineum* 580833 (Figures S1 and S2) [19,20,21].

### 3.4. Global Natural Product Social (GNPS) Molecular Networking

Aliquots (1 μL) of dried fraction (100 μg/mL in MeOH) were analysed on an Agilent 6545 Q-TOF LC/MS equipped with an Agilent 1290 Infinity II UPLC system, utilising an Agilent SB-C8 1.8 μm, 2.1 × 50 mm column, eluting with 90% H_2_O/MeCN to MeCN at a 0.417 mL/min over 2.5 min with an isocratic 0.1% formic acid modifier. UPLC-QTOF-(+)MS/MS data acquired for all samples at collision energy of 10, 20, and 40 eV were converted from Agilent MassHunter data files (.d) to mzXML file format using MSConvert software and transferred to the GNPS server (gnps.ucsd.edu). Molecular networking was performed using the GNPS data analysis workflow [22] using the spectral clustering algorithm with a cosine score of 0.6 and a minimum of 5 matched peaks. The resulting spectral network was imported into Cytoscape software (version 3.7.1) [23] and visualized using a ball-stick layout where nodes represent parent masses and the cosine score was reflected by edge thickness. Furthermore, group abundances were set as pie charts, which reflected the intensity of MS signals.

### 3.5. Extraction and Fractionation of an ISP-2 Agar Cultivation of CMB-MD14

The fungus *Penicillium* sp. CMB-MD14 was incubated on ISP-2 agar plates (×20) at 26.6 °C for 8 days, after which the diced agar was extracted with EtOAc (400 mL), filtered, and the organic phase concentrated in vacuo at 40 °C to yield an EtOAc extract (70 mg). The EtOAc extract was subjected to sequential solvent trituration (2 × 20 mL) to afford after concentration in vacuo *n*-hexane (21 mg) and MeOH (47 mg) solubles. The MeOH solubles were subjected to preparative reversed phase HPLC fractionation (PrepHT SB-C_8_ 5 μm, 21.2 × 150 mm column, 20 mL/min gradient elution over 20 min from 90% H_2_O/MeCN to MeCN inclusive of an isocratic 0.1% TFA modifier) to yield the anti-VRE active oxandrastin A (**1**) (2 mg, 2.8%). (see Scheme S1).

### 3.6. Extraction and Fractionation of a Rice Cultivation of CMB-MD14

A culture flask (2 L) containing medium grain rice (200 g) inoculated with the fungus CMB-MD14 was incubated at room temperature for 6 weeks, after which it was extracted with EtOAc (2 × 400 mL), and the combined organic phase filtered and concentrated in vacuo at 40 °C to yield the EtOAc extract (1.6 g). The EtOAc extract was subjected to solvent trituration (2 × 50 mL) to afford after concentration in vacuo *n*-hexane (850 mg) and MeOH (750 mg) solubles. A portion of the MeOH solubles (600 mg) was fractionated by preparative reversed phase HPLC (Phenomenex Luna-C_8_ 10 μm, 21.2 × 250 mm column, 20 mL/min gradient elution over 20 min from 90% H_2_O/MeCN to MeCN inclusive of an isocratic 0.1% TFA modifier) to yield fractions A–E (prioritized based on UPLC-DAD and UPLC-QTOF analyses). Fraction A (12.5 mg) was subjected to a semi-preparative reversed phase HPLC (ZORBAX SB-C_8_ 5 μm, 9.4 × 250 mm column, 3 mL/min gradient elution over 5 min from 90% H_2_O/MeCN to 60% H_2_O/MeCN, followed by isocratic elution over 30 min with 60% H_2_O/MeCN, inclusive of an isocratic 0.1% TFA/MeCN modifier) to yield isoaustalide F (**7**) (6.5 mg, 0.4%). A portion of fraction B (40 of 120 mg) was subjected to semi-preparative reversed phase HPLC (ZORBAX SB-Phenyl 5 μm, 9.4 × 250 mm column, 3 mL/min gradient elution over 20 min from 80% H_2_O/MeCN to 20% H_2_O/MeCN inclusive of an isocratic 0.1% TFA/MeCN modifier) to yield oxandrastin B (**2**) (26 mg, 4.87%). Fraction C (27 mg) was subjected to a semi-preparative reversed phase HPLC (ZORBAX SB-C_18_ 5 μm, 9.4 × 250 mm column, 3 mL/min isocratic elution over 21 min with 45% H_2_O/MeCN inclusive of an isocratic 0.1% TFA/MeCN modifier) to yield oxandrastin C (**3**) (13 mg, 0.81%). Fraction D (27 mg) was subjected to a semi-preparative reversed phase HPLC (ZORBAX SB-C_3_ 5 μm, 9.4 × 250 mm column, 3 mL/min isocratic elution over 35 min with 53% H_2_O/MeCN inclusive of an isocratic 0.1% TFA/MeCN modifier) to yield oxandrastin A (**1**) (19.3 mg, 1.2%) and andrastin F (**6**) (1.3 mg, 0.08%). Fraction E (14 mg) was subjected to a semi-preparative reversed phase HPLC (ZORBAX SB-Phenyl 5 μm, 9.4 × 250 mm column, 3 mL/min isocratic elution over 35 min with 47% H_2_O/MeCN inclusive of an isocratic 0.1% TFA/MeCN modifier) to yield andrastin C (**5**) (6 mg, 0.37%) (see Scheme S2). (Note: % yields estimated on a mass-to-mass basis against the weight of the EtOAc extract).

### 3.7. Metabolite Characterization

Oxandrastin A (**1**) White powder; [α]_D_ = −18.3 (*c* 0.44, MeOH) and [α]_D_ = −64 (*c* 0.51, CHCl_3_); NMR (CDCl_3_) see Table S1, Figures S7 and S8; NMR (methanol-*d*_4_) see Table 1, Table 2 and Table S2, Figures S10 and S11; ESI(+)MS *m*/*z* 531 [M+H]+; HRESI(+)MS *m*/*z* 553.2772 [M + Na]+ (calcd for C_30_H_42_O_8_Na, 553.2772).

Oxandrastin B (**2**) White powder; [α]_D_ = −39.8 (*c* 0.68, MeOH); NMR (methanol-*d*_4_) see Table 1, Table 2 and Table S3, Figures S13 and S14; ESI(+)MS *m*/*z* 489 [M+H]+; HRESI(+)MS *m*/*z* 511.2666 [M + Na]+ (calcd for C_28_H_40_O_7_Na, 511.2666).

Oxandrastin C (**3**) White powder; [α]_D_ = −24.7 (*c* 0.45, MeOH); NMR (methanol-*d*_4_) see Table 1, Table 2 and Table S4, Figures S16 and S17; ESI(+)MS *m*/*z* 489 [M+H]+; HRESI(+)MS *m*/*z* 511.2665 [M + Na]+ (calcd for C_28_H_40_O_7_Na, 511.2666).

Oxandrastin D (**4**) White powder; [α]_D_ = −15.1 (*c* 0.07, MeOH); NMR (methanol-*d*_4_) see Table 1, Table 2 and Table S5, Figures S19–S21; ESI(+)MS *m*/*z* 447 [M+H]+; HRESI(+)MS *m*/*z* 469.2561 [M + Na]+ (calcd for C_26_H_38_O_6_Na, 469.2561).

Andrastin C (**5**) White powder; [α]_D_ = −16.5 (*c* 0.45, MeOH) and [α]_D_ = −134.4 (*c* 0.38, CHCl_3_); NMR (methanol-*d*_4_) see Table S6, Figures S23 and S24; ESI(+)MS *m*/*z* 473 [M+H]+; HRESI(+)MS *m*/*z* 495.2722 [M + Na]+ (calcd for C_28_H_40_O_6_Na, 495.2717).

Andrastin F (**6**) White powder; [α]_D_ = −28.0 (*c* 0.10, MeOH) and [α]_D_ = −81.5 (*c* 0.09, CHCl_3_); NMR (methanol-*d*_4_) see Table S7, Figures S26 and S27; ESI(+)MS *m*/*z* 431 [M+H]+; HRESI(+)MS *m*/*z* 453.2613 [M + Na]+ (calcd for C_26_H_38_O_5_Na, 453.2611).

Isoaustalide F (**7**) White powder; [α]_D_ = −68.3 (*c* 0.30, MeOH) and [α]_D_ = −43 (*c* 0.38, CHCl_3_); NMR (methanol-*d*_4_) see Table 3 and Table S8, Figures S29 and S30; ESI(+)MS *m*/*z* 491 [M+H]+; HRESI(+)MS *m*/*z* 513.2095 [M + Na]+ (calcd for C_26_H_34_O_9_Na, 513.2095).

### 3.8. Hydrolysis of Oxandrastin B (2) to Oxandrastin D (4)

A mixture of **2** (2 mg) and K_2_CO_3_ (0.1 mg) in MeOH (1 mL) was stirred at room temperature for 12 h, with aliquots (2 μL) taken at 1, 4, and 12 h. Each aliquot was diluted with acetonitrile (200 μL) and analysed by UPLC-QTOF, to reveal complete conversion of **2** to **4** after 12 h. The 12 h hydrolysis reaction mixture was dried under N_2_, extracted with MeCN + 0.01% TFA modifier (2 mL) and purified by semi-preparative HPLC (ZORBAX SB-C_8_ 5 μm, 9.4 × 250 mm column, and 3 mL/min gradient elution over 20 min from 90% H_2_O/MeCN to MeCN, inclusive of an isocratic 0.1% TFA/MeCN modifier) to yield oxandrastin D (**4**) (0.8 mg) (Figures S4 and S5).

### 3.9. Antibiotic Assays

Antibacterial and antifungal assays were performed using prior published methods [24,25,26] as documented in the Supporting Information (Figure S37).

### 3.10. Cytotoxicity Assays

Cytotoxicity assays were performed using prior published methods [24,25,26] as documented in the Supporting Information (Figure S38).

## Figures and Tables

**Table 1:** Diagnostic ^1^H NMR (methanol-*d*_4_) data for oxandrastins A–D (**1**–**4**).

Position	δ_H_, Mult (*J* in Hz)			
	(1)	(2)	(3)	(4)
1	α. 1.82, dd (*12.3, 4.2*)	α. 1.81, dd (*12.3, 4.1*)	α. 1.72, dd (*12.0, 4.1*)	α. 1.71, dd (*12.0, 4.1*)
	β. 1.27, dd (*12.3, 12.3*)	β. 1.14, dd (*12.3, 12.1*)	β. 1.36, dd (*12.1, 12.0*)	β. 1.17, dd (*12.1, 12.0*)
2	5.25, ddd (*12.3, 4.2, 2.4*)	4.07, ddd (*12.1, 4.1, 2.8*)	5.17, ddd (*12.1, 4.1, 2.7*)	3.94, ddd (*11.9, 4.3, 3.0*)
3	4.96, d (*2.4*)	4.91, d (*2.8*)	3.44, d (*2.7*)	3.32, d (*3.0*) ^A^
5	1.40, dd (*12.1, 2.2*)	1.33, dd (*12.1, 2.9*)	1.45, dd (*12.1, 2.0*)	1.39, dd (*11.7, 2.7*)
6	α. 1.56, ddd (*13.3, 12.9, 3.0*)	α. 1.53, ddd (*12.8, 12.4, 3.1*)	α. 1.52, ddd (*13.1, 12.8, 3.1*)	α. 1.51, ddd (*12.8, 12.4, 3.2*)
	β. 1.52, m	β. 1.49, m	β. 1.46, m	β. 1.48, m
7	α. 2.13, br d (*13.4*)	α. 2.11, ddd (*12.8, 3.2, 3.1*)	α. 2.09, ddd (*13.1, 3.1, 2.4*)	α. 2.07, ddd (*13.2, 3.0, 2.8*)
	β. 2.77, m	β. 2.75, ddd (*12.8, 12.4, 5.0*)	β. 2.76, ddd (*13.1, 12.8, 4.4*)	β. 2.74, m
9	1.88, br s	1.85, br s	1.86, br s	1.82, br s
11	5.40, br s	5.44, br s	5.39, br s	5.44, br s
2-COCH_3_	1.92, s	-	2.03, s	-
3-COCH_3_	2.07, s	2.07, s	-	-
4-CH_3_ (α)	1.00, s	0.959, s	0.91, s	0.86, s
4-CH_3_ (β)	0.90, s	0.88, s	0.99, s	0.99, s
8-CH_3_	1.31, s	1.30, s	1.29, s	1.28, s
10-CH_3_	1.03, s	0.99, s	1.00, s	0.95, s
12-CH_3_	1.80, s	1.80, br s	1.78, br s	1.79, br s
13-CH_3_	1.18, s	1.18, s	1.17, s	1.17, s
14-CO_2_CH_3_	3.57, s	3.56, s	3.56, s	3.56, s
16-CH_3_	1.60, s	1.59, s	1.57, s	1.57, s

^A^ obscured by solvent signal.

**Table 2:** Diagnostic ^13^C NMR (methanol-*d*_4_) data for oxandrastins A–D (**1**–**4**).

Position	δ_C_, Type							
	(1)		(2)		(3)		(4)	
1	39.6	CH_2_	42.8	CH_2_	38.8	CH_2_	42.2	CH_2_
2	69.4	CH	65.6	CH	71.9	CH	67.0	CH
3	78.5	CH	81.6	CH	77.2	CH	79.7	CH
4	39.3	C	39.4	C	40.0	C	39.6	C
5	50.0	CH	49.9	CH	48.4	CH	48.3	CH
6	18.6	CH_2_	18.6	CH_2_	18.7	CH_2_	18.8	CH_2_
7	34.1	CH_2_	34.1	CH_2_	34.2	CH_2_	34.3	CH_2_
8	43.5	C	43.5	C	43.5	C	43.6	C
9	54.5	CH	54.6	CH	54.6	CH	54.6	CH
10	39.5	C	39.4	C	39.4	C	39.2	C
11	125.6	CH	125.9	CH	126.0	CH	126.4	CH
12	137.2	C	136.9	C	136.7	C	136.3 ^a^	C
13	58.6 ^a^	C	58.4 ^a^	C	58.4	C	58.8 ^a^	C
14	68.9 ^a^	C	68.9 ^a^	C	68.8	C	68.8 ^a^	C
15	ND	C	ND	C	ND	C	ND	C
16	114.8	C	114.6	C	114.8	C	114.9 ^a^	C
17	ND	C	201.8 ^a^	C	202.3 ^a^	C	ND	C
2-COCH_3_	172.3	C	-	-	172.7	C	-	-
2-COCH_3_	21.1	CH_3_	-	-	21.3	CH_3_	-	-
3-COCH_3_	172.6	C	173.1	C	-	-	-	-
3-COCH_3_	20.9	CH_3_	21.2	CH_3_	-	-	-	-
4-CH_3_ (α)	21.7	CH_3_	21.9	CH_3_	22.0	CH_3_	22.1	CH_3_
4-CH_3_ (β)	28.2	CH_3_	28.3	CH_3_	29.1	CH_3_	29.2	CH_3_
8-CH_3_	18.3	CH_3_	18.4	CH_3_	18.3	CH_3_	18.4	CH_3_
10-CH_3_	18.4	CH_3_	18.5	CH_3_	18.5	CH_3_	18.7	CH_3_
12-CH_3_	19.9	CH_3_	19.9	CH_3_	19.9	CH_3_	19.9	CH_3_
13-CH_3_	16.2	CH_3_	16.2	CH_3_	16.2	CH_3_	16.2	CH_3_
14-CO_2_CH_3_	172.0	C	172.0	C	172.1	C	172.1	C
14-CO_2_CH_3_	52.2	CH_3_	52.2	CH_3_	52.1	CH_3_	52.1	CH_3_
16-CH_3_	6.4	CH_3_	6.4	CH_3_	6.4	CH_3_	6.4	CH_3_

ND not detected; ^a^ resonances detected from HMBC data.

**Table 3:** Diagnostic ^1^H and ^13^C NMR (methanol-*d*_4_) data for isoaustalide F (**7**).

Position	δ_H_, Mult (*J* in Hz)	δ_C_	Type
1	5.21, br s	69.9	CH_2_
2	-	171.9	C
3	-	108.4	C
4	-	156.7	C
5	-	117.5	C
6	-	160.0	C
7	-	116.2	C
8	-	147.6	C
9	-	77.6	C
10	a. 2.33, dd (*15.7, 2.0*)	43.4	CH_2_
	b. 2.16, dd (*15.7, 4.3*)		
11	4.16, dd (*3.9, 2.0*)	69.7	CH
12	-	87.5	C
13	-	87.0	C
14	-	120.1	C
15	3.71, dd (*10.5, 5.9*)	68.8	CH
16	a. 2.18, dd (*13.9, 5.9*)	40.5	CH_2_
	b. 1.68, dd (*13.9, 10.5*)		
17	-	44.6	C
18	2.40, d (*8.1*)	39.4	CH
19	a. 3.01, d (*18.6*)	19.2	CH_2_
	b. 2.94, dd (*18.6, 8.1*)		
7-CH_3_	2.07, s	10.8	CH_3_
9-CH_3_	1.23, s	28.3	CH_3_
13-CH_3_(a)	1.52, s	29.3	CH_3_
13-CH_3_(b)	1.63, s	26.7	CH_3_
17-CH_3_	1.03, s	18.8	CH_3_
4-OCH_3_	4.06, s	62.4	CH_3_
14-OCH_3_	3.41, s	49.3 ^A^	CH_3_

^A^ resonance obscured by residue solvent.

## Data Availability

Data is contained within the article or Supplementary Materials.

## Supplementary Materials

The following are available online, Fungal taxonomy, NMR spectra and tabulated data for **1**–**7**, bioassay methods and results, and GNPS analyses.

## Sample Availability

Not available.
