# Supplementary material for: Oxandrastins: Antibacterial Meroterpenes from an Australian Mud Dauber Wasp Nest-Associated Fungus, Penicillium sp. CMB-MD14
Source: Molecules. 2021 Nov 25;26(23):7144. doi: 10.3390/molecules26237144 (PMC8659017; doi:10.3390/molecules26237144)
Supplement: Supplementary file 1 [file molecules-26-07144-s001.zip › molecules-1478023-supplementary.pdf]

Supplementary Material

Oxandrastins: Antibacterial meroterpenes from an Australian mud-dauber wasp nest-associated fungus, *Penicillium* sp. CMB-MD14

Ahmed H. Elbanna,[†,§] Zeinab G. Khalil[†] and Robert J. Capon[†*]

[†] Institute for Molecular Bioscience, The University of Queensland, Brisbane, QLD 4072, Australia

[§] Current address: Department of Pharmacognosy, Faculty of Pharmacy, Cairo University, Cairo 11562, Egypt

*Corresponding author r.capon@uq.edu.au

Table of content

Table of content ... ii

List of figures ... iii

List of tables ... v

List of schemes ... v

1 Experimental ... 1

1.1.1 ITS gene sequence of CMB-MD22 ... 1

1.1.2 BLAST search (1st closest match) ... 1

1.1.3 Phylogenetic tree ... 2

1.2 Cultivation of *Penicillium sp.* CMB-MD14 on different media ... 3

1.3 Extraction and fractionation of an ISP-2 agar cultivation of CMB-MD14 ... 4

1.4 Extraction and fractionation of a rice cultivation of CMB-MD14 ... 4

2 Analysis of the hydrolysis of oxandrastin B (**2**) to oxandrastin D (**4**) ... 5

3 Spectroscopic characterization of metabolites ... 6

3.1.1 Oxandrastin A (**1**) ... 6

3.1.2 Oxandrastin B (**2**) ... 10

3.1.3 Oxandrastin C (**3**) ... 12

3.1.4 Oxandrastin D (**4**) ... 14

3.1.5 Andrastin C (**5**) ... 17

3.1.6 Andrastin F (**6**) ... 19

3.1.7 Isoaustalide F (**7**) ... 21

4 GNPS molecular networking analysis ... 24

5 Biological screening ... 26

5.1 Anti-microbial ... 26

5.2 Cytotoxicity assays ... 27

List of Figures

Figure S1. Blast search (closest match) for CMB-MD14..2

Figure S2. Phylogenetic tree by PhyML Maximum Likehood analysis of 18s rRNA sequence showing the relationship of CMB-MD14 among selected reference strains (RefSeq GenBank) with their accession numbers. 3

Figure S3. Representative UPLC-DAD chromatograms (210 nm) of culture condition optimization for production of oxandrastins from CMB-MD14 cultivated on different solid based media and UV-vis spectra of andrastins/oxandrastins (red) and austalides (blue). 3

Figure S4. UPLC-QTOF chromatogram for hydrolysis of 2 to yield 4 (red peaks). a) CMB-MD14 rice crude extract; b) SIE for 4 (*m/z* $[M+H]^+$ 447) in the rice crude extract; c-e) hydrolysis of 2 over 1, 4 h and 12 h, respectively. 5

Figure S5. UPLC-QTOF MSMS fragmentation of a) *m/z* 447 detected in CMB-MD14 rice crude extract by SIE feature and b) oxandrastin D (4). 5

Figure S6. Key 2D NMR (600 MHz, $CDCl_3$) correlations for oxandrastin A (1). 6

Figure S7. ^{1}H NMR (600 MHz, $CDCl_3$) spectrum for oxandrastin A (1). 7

Figure S8. ^{13}C NMR (150 MHz, $CDCl_3$) spectrum for oxandrastin A (1). 7

Figure S9. Key 2D NMR (600 MHz, MeOH-d_4) correlations for oxandrastin A (1). 8

Figure S10. ^{1}H NMR (600 MHz, MeOH-d_4) spectrum for oxandrastin A (1). 9

Figure S11. ^{13}C NMR (150 MHz, MeOH-d_4) spectrum for oxandrastin A (1). 9

Figure S12. Key 2D NMR (600 MHz, MeOH-d_4) correlations for oxandrastin B (2). 10

Figure S13. ^{1}H NMR (600 MHz, MeOH-d_4) spectrum for oxandrastin B (2)........................11

Figure S14. ^{13}C NMR (150 MHz, MeOH-d_4) spectrum for oxandrastin B (2). 11

Figure S15. Key 2D NMR (600 MHz, MeOH-d_4) correlations for oxandrastin C (3). 12

Figure S16. ^{1}H NMR (600 MHz, MeOH-d_4) spectrum for oxandrastin C (3)........................13

Figure S17. ^{13}C NMR (150 MHz, MeOH-d_4) spectrum for oxandrastin C (3). 13

Figure S18. Key 2D NMR (600 MHz, MeOH-d_4) correlations for oxandrastin D (4). 14

Figure S19. ^{1}H NMR (600 MHz, MeOH-d_4) spectrum for oxandrastin D (4). 15

Figure S20. ^{13}C NMR (150 MHz, MeOH-d_4) spectrum for oxandrastin D (4). 15

Figure S21. ^{1}H NMR (600 MHz, MeOH-d_4) spectra for oxandrastins a) A (1), b) B (2), c) C (3) and d) D (4). 16
Figure S22. Key 2D NMR (600 MHz, MeOH-d_4) correlations for andrastin C (5). 17
Figure S23. ^{1}H NMR (600 MHz, MeOH-d_4) spectrum for andrastin C (5)....18
Figure S24. ^{13}C NMR (150 MHz, MeOH-d_4) spectrum for andrastin C (5). 18
Figure S25. Key 2D NMR (600 MHz, MeOH-d_4) correlations for andrastin F (6). 19
Figure S26. ^{1}H NMR (600 MHz, MeOH-d_4) spectrum for andrastin F (6). 20
Figure S27. ^{13}C NMR (150 MHz, MeOH-d_4) spectrum for andrastin F (6). 20
Figure S 28. Key 2D NMR (600 MHz, MeOH-d_4) correlations for isoaustalide F (7). 21
Figure S29. ^{1}H NMR (600 MHz, MeOH-d_4) spectrum for isoaustalide F (7). 22
Figure S30. ^{13}C NMR (150 MHz, MeOH-d_4) spectrum for isoaustalide F (7). 22
Figure S31. Energy-minimized (MM2) structure of isoaustalide F (7) and Key ROESY NMR (600 MHz, MeOH-d_4) correlations. 23
Figure S32. Experimental CD spectrum of a) isoaustalide F (7), 0.05% in acetonitrile compared to b) reported ECD spectra of austalides A (black), J (green), T (red) and U (blue) in acetonitrile (figure extracted from publication).[4] 23
Figure S33. GNPS molecular network for CMB-MD14 rice culture. Red circle represents andrastins and oxandrastins cluster, and green circle represents austalides cluster. 24
Figure S34. GNPS cluster of oxandrastins and andrastins in CMB-MD14 rice culture with the authentic compounds (1–6). 24
Figure S35. GNPS cluster of austalides in in CMB-MD14 rice culture with the authentic compound 7. 25
Figure S36. UPLC-QTOF analysis of austalides in CMB-MD14 rice crude extract.. 25
Figure S37. Anti-microbial activities of metabolites 1–7. 27
Figure S38. Cytotoxicity of metabolites 1–7. 28

List of tables

Table S1. 1D and 2D NMR (600 MHz, $CDCl_3$) data for oxandrastin A (1) 6

Table S2. 1D and 2D NMR (600 MHz, MeOH-d_4) data for oxandrastin A (1) 8

Table S3. 1D and 2D NMR (600 MHz, MeOH-d_4) data for oxandrastin B (2).......................10

Table S4. NMR (600 MHz, MeOH-d_4) data for oxandrastin C (3) 12

Table S5. NMR (600 MHz, MeOH-d_4) data for oxandrastin D (4) 14

Table S6. 1D and 2D NMR (600 MHz, MeOH-d_4) data for andrastin C (5)...........................17

Table S7. 1D and 2D NMR (600 MHz, MeOH-d_4) data for andrastin F (6) 19

Table S8. 1D and 2D NMR (600 MHz, MeOH-d_4) data for isoaustalide F (7).......................21

List of schemes

Scheme S1: Isolation scheme of metabolite 1 from ISP-2 agar culture; a) trituration of crude extract with hexane (-1) yielding defatted crude extract (-2); b) preparative HPLC fractionation. 4

Scheme S2: Isolation scheme of metabolites 1–7; a) trituration of crude extract with hexane (-1) yielding defatted crude extract (-2); b) preparative HPLC fractionation; c) semi-preparative HPLC purification; d) semi-preparative HPLC purification for 40 mg; e) hydrolysis for 2 mg followed by semi-preparative HPLC purification...4

1 Experimental

1.1.1 ITS gene sequence of CMB-MD22

CCGAGGTCACCTGGAAGATTGATTGGGGTCGCCGGCGGGCGCCGGCCGGGCCTACAGAGCGGGTG
ACGAAGCCCCATACGCTCGAGGACCGGACGCGGTGCCGCCGCTGCCTTTCGGGCCCGCCCCCCGGG
AGCCGGGGGGCGAAGCCCAACACACAAGCCGTGCTTGAGGGCAGCAATGACGCTCGGACAGGCAT
GCCCCCCGGAATACCAGGGGGCGCAATGTGCGTTCAAAGACTCGATGATTCACTGAATTCTGCAATT
CACATTACTTATCGCATTTCGCTGCGTTCTTCATCGATGCCGGAACCAAGAGATCCGTTGTTGAAAGT
TTTAACTGATTTAGCTAATCTACTCAGACTGCAATCTTCAGACAGAGTTCAATGGTGTCTTCGGCGGG
CGCGGGCCCGGGGGCGGATGCCCCCCGGCGGCCGTGAGGCGGGCCCGCCGAAGCAACAAGGTAC
GATAAACACGGGTGGGAGGTTGGACCCAGAGGGCCCTCACTCGGTAATGATCCTTCCGCAGGTTCA
CCTTACGGAA

1.1.2 BLAST search (1st closest match)

Penicillium panissanguineum isolate 580833 small subunit ribosomal RNA gene, partial sequence; internal transcribed spacer 1, 5.8S ribosomal RNA gene, and internal transcribed spacer 2, complete sequence; and large subunit ribosomal RNA gene, partial sequence

Sequence ID: MK387978.1 Length: 577 Number of Matches: 1

Range 1: 1 to 540 GenBank Graphics ▼ Next Match ▲ Previous Match

Score	Expect	Identities	Gaps	Strand
963 bits(521)	0.0	535/541(99%)	3/541(0%)	Plus/Minus

```
Query  1    CCGAGGTC-ACCTGG-AAGATTGATTGGGGTCGCCGGCGGGCGCCGGCCGGGCCTACAGA  58
            |||||||| |||||| ||||||||||||||||||||||||||||||||||||||||||||
Sbjct  540  CCGAGGTCAACCTGGAAAGATTGATTGGGGTCGCCGGCGGGCGCCGGCCGGGCCTACAGA  481

Query  59   GCGGGTGACGAAGCCCCATACGCTCGAGGACCGGACGCGGTGCCGCCGCTGCCTTTCGGG  118
            ||||||||| ||||||||||||||||||||||||||||||||||||||||||||||||||
Sbjct  480  GCGGGTGACAAAGCCCCATACGCTCGAGGACCGGACGCGGTGCCGCCGCTGCCTTTCGGG  421

Query  119  CCCGCCCCCCGGGAGCCGGGGGGCGAAGCCCAACACACAAGCCGTGCTTGAGGGCAGCAA  178
            ||||||||||||||||||||||||||||||||||||||||||||||||||||||||||||
Sbjct  420  CCCGCCCCCCGGGAGCCGGGGGGCGAAGCCCAACACACAAGCCGTGCTTGAGGGCAGCAA  361

Query  179  TGACGCTCGGACAGGCATGCCCCCCGGAATACCAGGGGGCGCAATGTGCGTTCAAAGACT  238
            ||||||||||||||||||||||||||||||||||||||||||||||||||||||||||||
Sbjct  360  TGACGCTCGGACAGGCATGCCCCCCGGAATACCAGGGGGCGCAATGTGCGTTCAAAGACT  301

Query  239  CGATGATTCACTGAATTCTGCAATTCACATTACTTATCGCATTTCGCTGCGTTCTTCATC  298
            ||||||||||||||||||||||||||||||||||||||||||||||||||||||||||||
Sbjct  300  CGATGATTCACTGAATTCTGCAATTCACATTACTTATCGCATTTCGCTGCGTTCTTCATC  241

Query  299  GATGCCGGAACCAAGAGATCCGTTGTTGAAAGTTTTAACTGATTTAGCTAATCTACTCAG  358
            |||||||||||||||||||||||||||||||||||||||||||||||||||||| |||||
Sbjct  240  GATGCCGGAACCAAGAGATCCGTTGTTGAAAGTTTTAACTGATTTAGCTAATCTGCTCAG  181

Query  359  ACTGCAATCTTCAGACAGAGTTCAATGGTGTCTTcggcgggcgcgggcccgggggcggat  418
            |||||||||||||||||||||||||||||||||||||||||||||||||||||||||| |
Sbjct  180  ACTGCAATCTTCAGACAGAGTTCAATGGTGTCTTCGGCGGGCGCGGGCCCGGGGGCGGGT  121

Query  419  gccccccggcggccgtgaggcgggcccgccgAAGCAACAAGGTACGATAAACACGGGTGG  478
            ||||||||||||||||||||||||||||||||||||||||||||||||||||||||||||
Sbjct  120  GCCCCCCGGCGGCCGTGAGGCGGGCCCGCCGAAGCAACAAGGTACGATAAACACGGGTGG  61

Query  479  GAGGTTGGACCCAGAGGGCCCTCACTCGGTAATGATCCTTCCGCAGGTTCACCTTACGGA  538
            |||||||||||||||||||||||||||||||||||||||||||||||||||||| |||||
Sbjct  60   GAGGTTGGACCCAGAGGGCCCTCACTCGGTAATGATCCTTCCGCAGGTTCACCT-ACGGA  2

Query  539  A  539
            |
Sbjct  1    A  1
```

Penicillium panissanguineum isolate 580833 small subunit ribosomal RNA gene, partial sequence; internal transcribed spacer 1, 5.8S ribosomal RNA gene, and internal transcribed spacer 2, complete sequence; and large subunit ribosomal RNA gene, partial sequence

GenBank: MK387978.1

FASTA Graphics

Go to:

```
LOCUS       MK387978                 577 bp    DNA     linear   PLN 15-JAN-2019
DEFINITION  Penicillium panissanguineum isolate 580833 small subunit ribosomal
            RNA gene, partial sequence; internal transcribed spacer 1, 5.8S
            ribosomal RNA gene, and internal transcribed spacer 2, complete
            sequence; and large subunit ribosomal RNA gene, partial sequence.
ACCESSION   MK387978
VERSION     MK387978.1
KEYWORDS    .
SOURCE      Penicillium panissanguineum
  ORGANISM  Penicillium panissanguineum
            Eukaryota; Fungi; Dikarya; Ascomycota; Pezizomycotina;
            Eurotiomycetes; Eurotiomycetidae; Eurotiales; Aspergillaceae;
            Penicillium.
REFERENCE   1  (bases 1 to 577)
  AUTHORS   Cheng,K. and Yu,S.
  TITLE     Host specificity of root-associated fungi of different species in
            subtropical forest
  JOURNAL   Unpublished
REFERENCE   2  (bases 1 to 577)
  AUTHORS   Cheng,K. and Yu,S.
  TITLE     Direct Submission
  JOURNAL   Submitted (08-JAN-2019) School Of Life Sciences, Sun Yat-sen
            University, No. 135, Xingang Xi Road, Guangzhou, Guangdong 510275,
            China
```

Figure S1. Blast search (closest match) for CMB-MD14

1.1.3 Phylogenetic tree

Phylogenetic tree obtained by PhyML Maximum Likelihood analysis was constructed using the top similar 18S rRNA sequences displayed after BLAST on Refseq RNA NCBI database using CMB-MD14 18S rRNA as queries. The JC69 model was used to infer phylogeny sequences.[24] Sequences alignments were produced with the MUSCLE program.[25] Phylogenetic tree was constructed using the UGENE program using the aforementioned models and visualized using Ugene's tree view.[26]

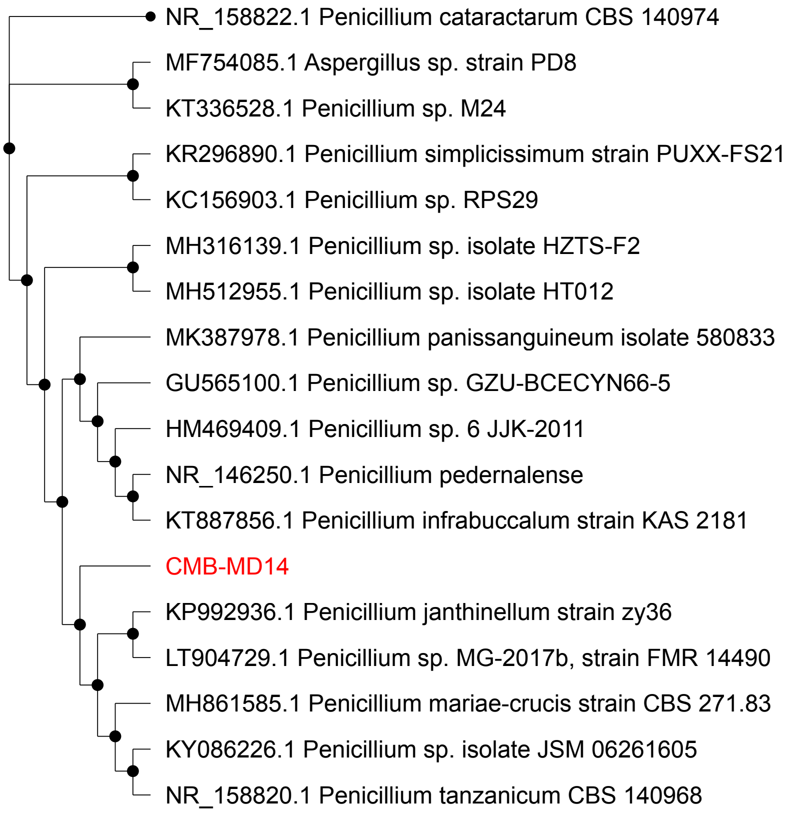

Figure S2. Phylogenetic tree by PhyML Maximum Likehood analysis of 18s rRNA sequence showing the relationship of CMB-MD14 among selected reference strains (RefSeq GenBank) with their accession numbers.

1.2 Cultivation of *Penicillium sp.* CMB-MD14 on different media

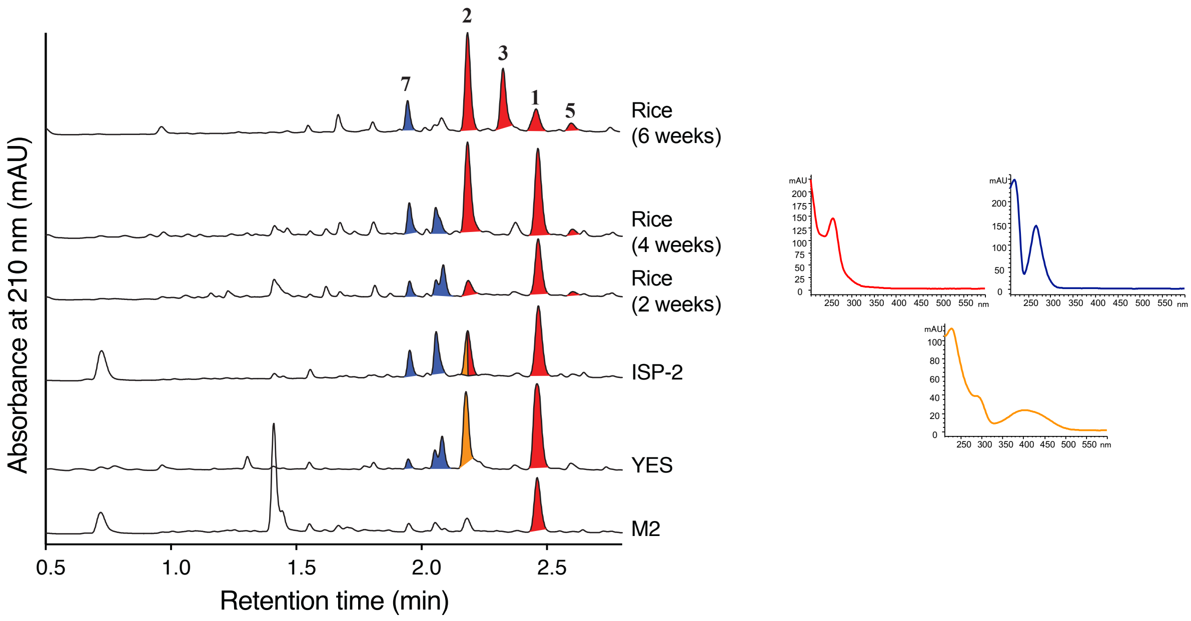

Figure S3. Representative UPLC-DAD chromatograms (210 nm) of culture condition optimization for production of oxandrastins from CMB-MD14 cultivated on different solid based media and UV-vis spectra of andrastins/oxandrastins (red) and austalides (blue).

1.3 Extraction and fractionation of an ISP-2 agar cultivation of CMB-MD14

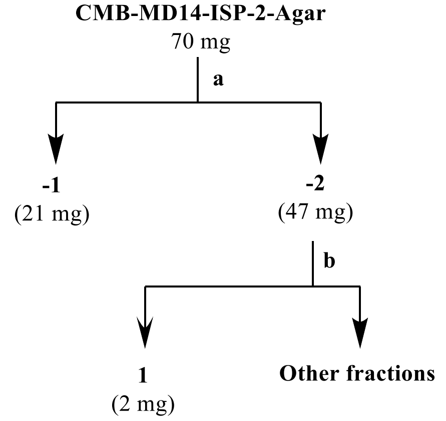

Scheme S1: Isolation scheme of metabolite 1 from ISP-2 agar culture; a) trituration of crude extract with hexane (-1) yielding defatted crude extract (-2); b) preparative HPLC fractionation.

1.4 Extraction and fractionation of a rice cultivation of CMB-MD14

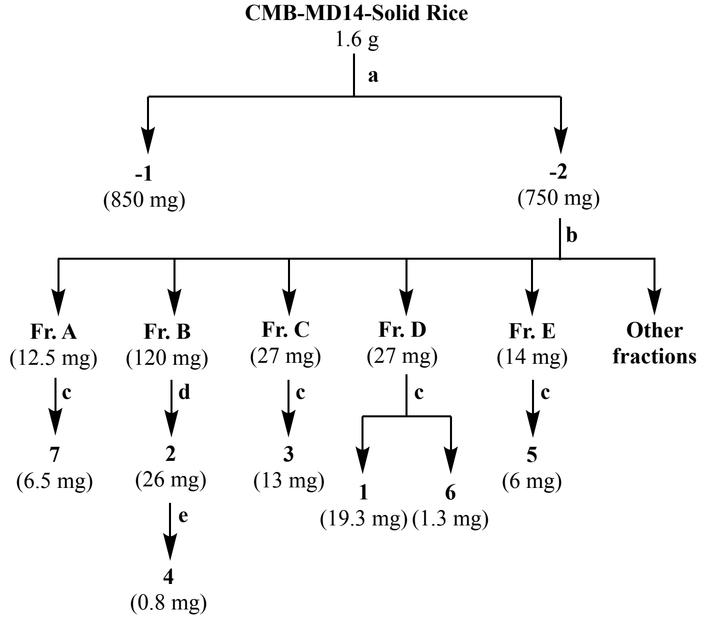

Scheme S2: Isolation scheme of metabolites **1–7**; **a)** trituration of crude extract with hexane (-1) yielding defatted crude extract (-2); **b)** preparative HPLC fractionation; **c)** semi-preparative HPLC purification; **d)** semi-preparative HPLC purification for 40 mg; **e)** hydrolysis for 2 mg followed by semi-preparative HPLC purification.

2 Analysis of the hydrolysis of oxandrastin B (2) to oxandrastin D (4)

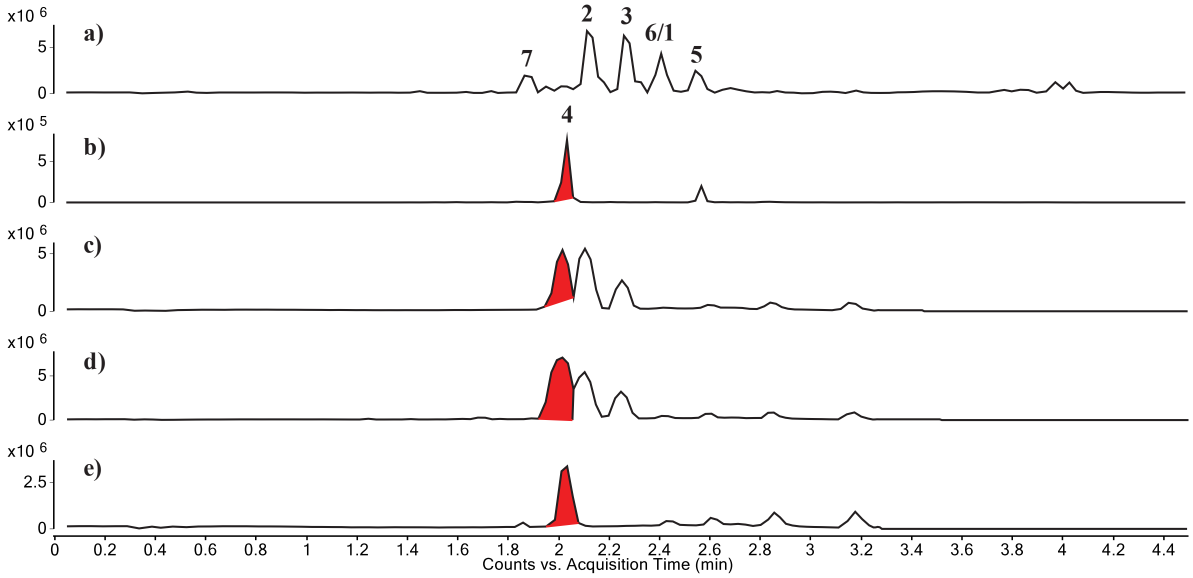

Figure S4. UPLC-QTOF chromatogram for hydrolysis of **2** to yield **4** (red peaks). a) CMB-MD14 rice crude extract; b) SIE for **4** (*m/z* $[M+H]^+$ 447) in the rice crude extract; c-e) hydrolysis of **2** over 1, 4 h and 12 h, respectively.

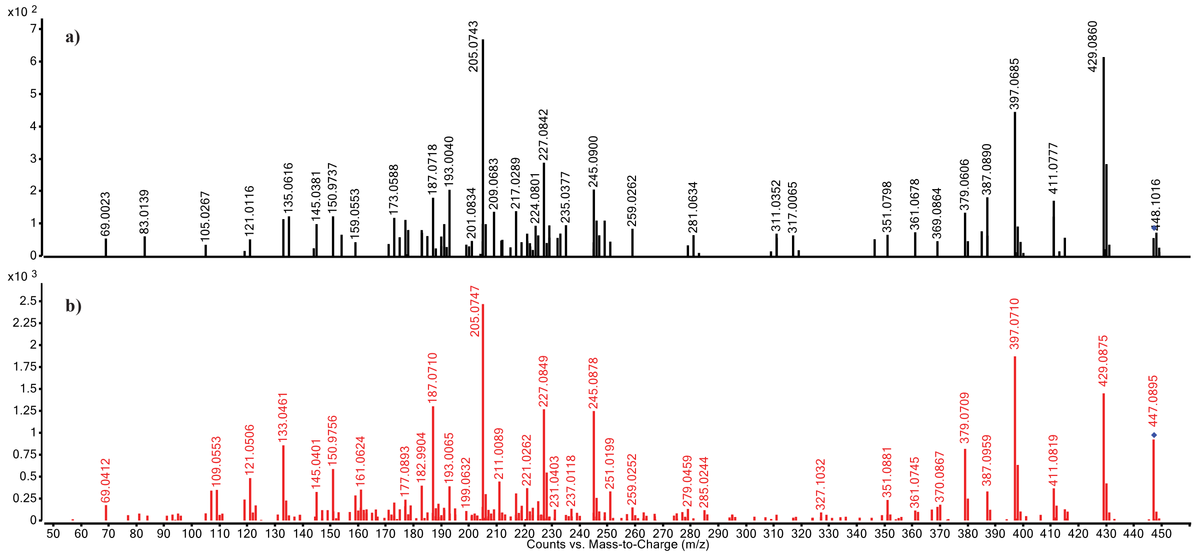

Figure S5. UPLC-QTOF MSMS fragmentation of a) *m/z* 447 detected in CMB-MD14 rice crude extract by SIE feature and b) oxandrastin D (**4**).

3 Spectroscopic characterization of metabolites

3.1.1 Oxandrastin A (1)

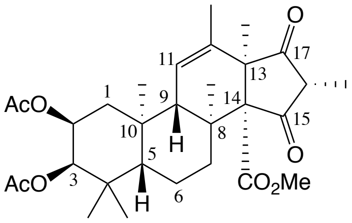

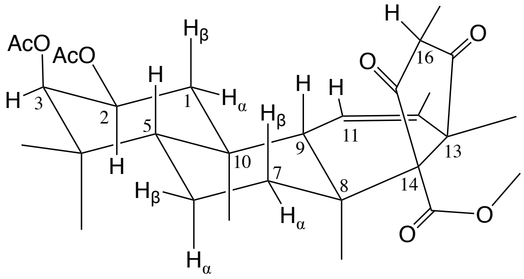

Table S1. 1D and 2D NMR (600 MHz, $CDCl_3$) data for oxandrastin A (**1**)

Position	δ_H, mult (*J* in Hz)	δ_C	COSY	^{1}H-^{13}C HMBC	ROESY
1	α 1.81, dd (*12.2, 4.0*)	38.5	1β, 2	2, 3, 5, 10	2, 10-CH_3
	β 1.27, dd (*12.4, 12.2*)		1α, 2		9
2	5.24, ddd (*12.4, 4.0, 3.1*)	68.0	1, 3	2-CO	1α, 4-CH_3 (α), 10-CH_3
3	4.96, br s	76.9[a]	2	1, 2, 5, 3-CO, 4-CH_3 (β)	4-CH_3 (α)
4	---	38.3	---	---	---
5	1.31, dd (*11.1, 3.3*)	48.7	6	---	7β, 9
6	1.52, m	17.3	7	5, 7, 8, 10	
7	α 2.28, br d (*13.4*)	31.8	6, 7β	5, 8, 9	8-CH_3
	β 2.55, ddd (*12.6, 12.6, 4.9*)		6, 7α	8, 8-CH_3	5, 9
8	---	40.4	---		---
9	1.84, br s	53.7	11	8, 10, 11, 12, 14, 8-CH_3/10-CH_3	1β, 5, 7β, 16
10	---	38.7	---		---
11	5.58, br s	126.8	9, 12-CH_3	8, 9, 10, 13, 12-CH_3	1α, 10-CH_3, 12-CH_3
12	---	135.1	---	---	---
13	---	61.0	---	---	---
14	---	73.2	---	---	---
15	---	210.7	---	---	---
16	3.21, q (*6.8*)	51.1	16-CH_3	15, 17, 16-CH_3	9, 12-CH_3
17	---	209.5	---	---	---
2-CO	---	170.6	---	---	---
2-COCH_3	1.94, s	21.2	---	2-CO	
3-CO	---	171.0	---	---	---
3-COCH_3	2.12, s	21.3	---	3-CO	
4-CH_3 (α)	0.97, s	21.4	---	3, 5, 4-CH_3 (β)	2, 3
4-CH_3 (β)	0.86, s	27.6	---	3, 4, 5, 4-CH_3 (α)	
8-CH_3	1.23, s	18.1[b]	---	7, 8, 9, 14	
10-CH_3	0.98, s	18.1[b]	---	1, 5, 9	
12-CH_3	1.68, s	19.2	11	11, 12, 13	11, 13-CH_3
13-CH_3	1.27, s	16.5	---	12, 13, 14, 17	12-CH_3
14-CO_2	---	168.4	---	---	---
14-CO_2CH_3	3.54, s	52.0	---	14-CO_2	13-CH_3, 16-CH_3
16-CH_3	1.19, d (*6.8*)	9.6	16	15, 16, 17	

(a) obscured by solvent signal, (b) signals are interchangeable

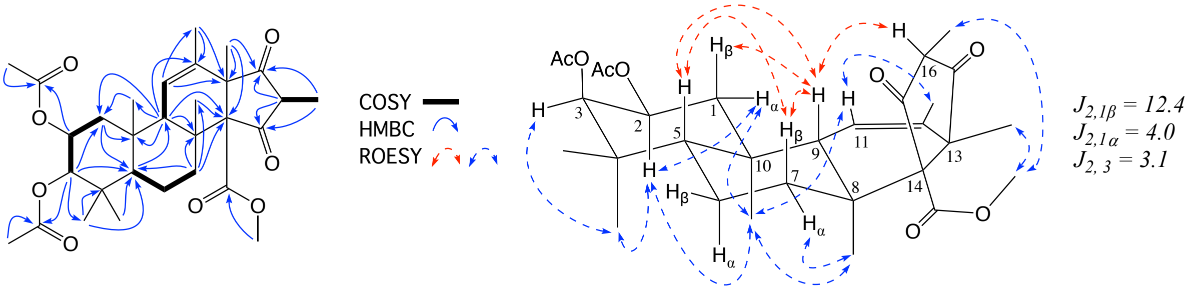

Figure S6. Key 2D NMR (600 MHz, $CDCl_3$) correlations for oxandrastin A (**1**).

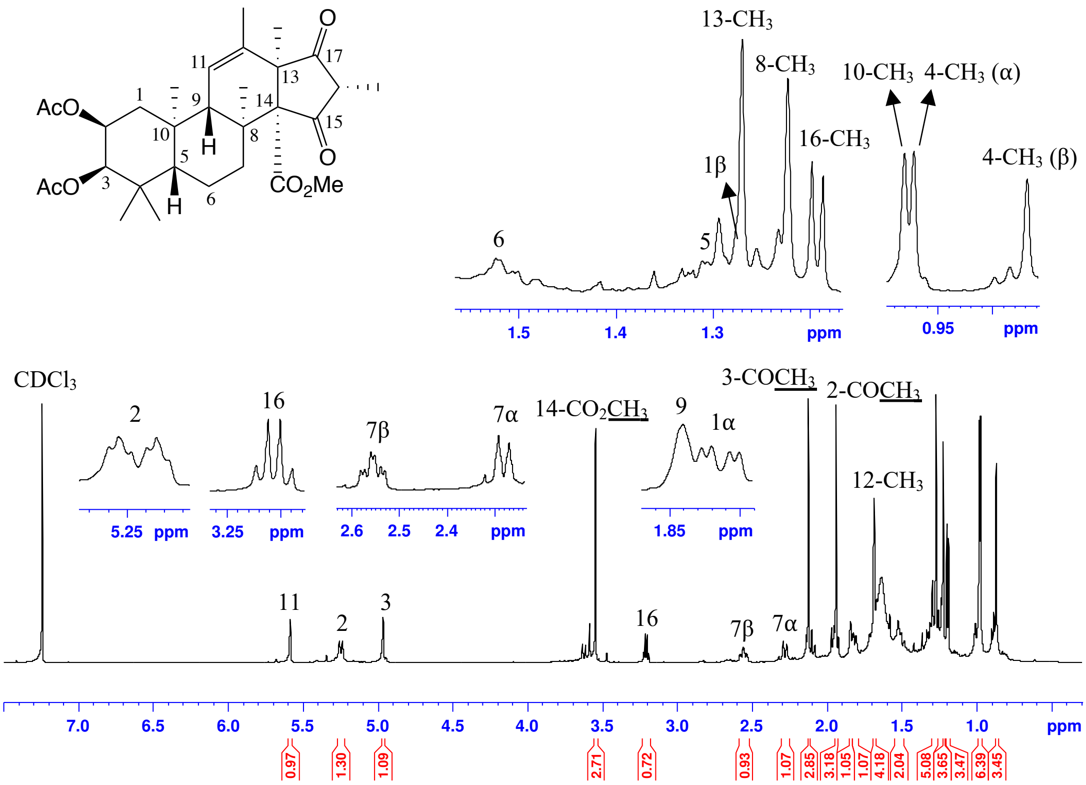

Figure S7. ^{1}H NMR (600 MHz, CDCl$_3$) spectrum for oxandrastin A (**1**).

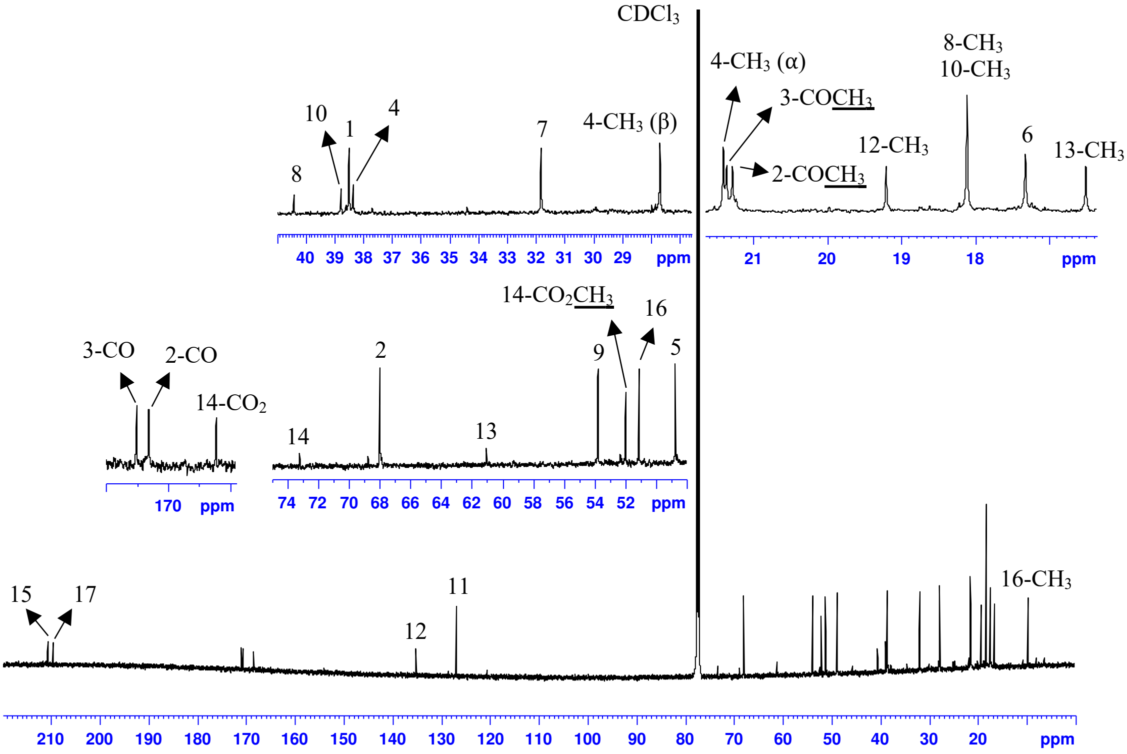

Figure S8. ^{13}C NMR (150 MHz, CDCl$_3$) spectrum for oxandrastin A (**1**).

NMR data acquired in MeOH indicates a tautomerism in the 5-membered ring (absence of 16-H, C-15 and C-17 resonances along with appearance of an olefinic singlet 16-CH_3).

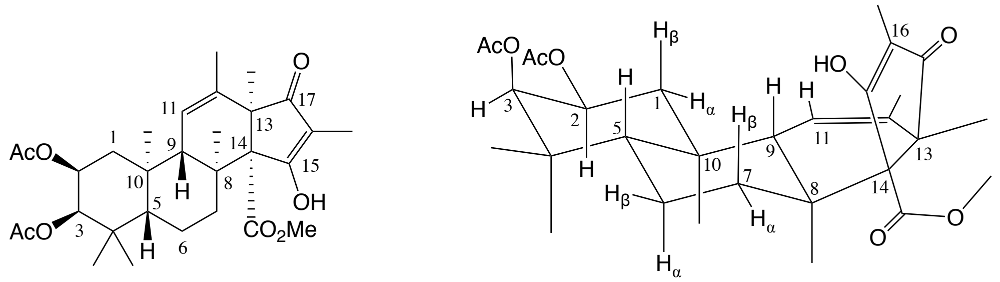

Table S2. 1D and 2D NMR (600 MHz, MeOH-*d*$_4$) data for oxandrastin A (**1**)

**Position**	δ_H, mult (*J* in Hz)	δ_C	COSY	^{1}H-^{13}C HMBC	ROESY
1	α 1.82, dd (*12.3, 4.2*)	39.6	1β, 2	2, 3, 5, 10, 10-CH_3	2, 10-CH_3, 11
	β 1.27, dd (*12.3, 12.3*)		1α, 2	2, 3, 9, 10, 10-CH_3	9
2	5.25, ddd (*12.3, 4.2, 2.4*)	69.4	1, 3	1, 3, 2-CO	1α, 3, 4-CH_3 (α), 10-CH_3
3	4.96, d (*2.4*)	78.5	2	1, 2, 4, 5, 3-CO, 4-CH_3 (α/β)	2, 4-CH_3 (α)
4	---	39.3	---	---	---
5	1.40, dd (*12.1, 2.2*)	50.0	6	1/4/10, 7, 9, 4-CH_3 (α/β), 10-CH_3	1β, 4-CH_3 (β), 7β, 9
6	α 1.56, ddd (*13.3, 12.9, 3.0*)	18.6	7α, 5	5, 7, 8, 10	4-CH_3 (α), 8-CH_3
	β 1.52, m		5	5, 7, 8, 10	4-CH_3 (β)
7	α 2.13, br d (*13.4*)	34.1	6, 7β	5, 8, 9, 8-CH_3	8-CH_3
	β 2.77, m		6, 7α	---	5, 9
8	---	43.5	---	---	---
9	1.88, br s	54.5	11	8, 10, 11, 12, 14, 8-CH_3, 10-CH_3	1β, 5, 7β
10	---	39.5	---	---	---
11	5.40, br s	125.6	9, 12-CH_3	8, 9, 10, 13, 12-CH_3	1α, 10-CH_3, 12-CH_3
12	---	137.2	---	---	---
13	---	58.6 [a]	---	---	---
14	---	68.9 [a]	---	---	---
15	---	**ND***	---	---	---
16	---	114.8	---	---	---
17	---	**ND***	---	---	---
2-CO	---	172.3	---	---	---
2-COCH$_3$	1.92, s	21.1	---	2, 2-CO	
3-CO	---	172.6	---	---	---
3-COCH$_3$	2.07, s	20.9	---	3, 3-CO	
4-CH_3 (α)	1.00, s	21.7	---	3, 4, 5, 4-CH_3 (β)	2, 3, 6α
4-CH_3 (β)	0.90, s	28.2	---	3, 4, 5, 4-CH_3 (α)	3, 5, 6β
8-CH_3	1.31, s	18.3	---	7, 8, 9, 14	6α, 7α, 10-CH_3
10-CH_3	1.03, s	18.4	---	1/10, 5, 9	1α, 2, 6α, 11, 8-CH_3
12-CH_3	1.80, s	19.9	11	11, 12	11, 13-CH_3
13-CH_3	1.18, s	16.2	---	12, 13, 14	12-CH_3
14-CO_2CH_3	---	172.0	---	---	---
14-CO_2CH_3	3.57, s	52.2	---	14-CO_2	13-CH_3
16-CH_3	1.60, s	6.4	---	16	---

(a) values determined from ^{13}C NMR and HMBC spectra, ***ND** not detected

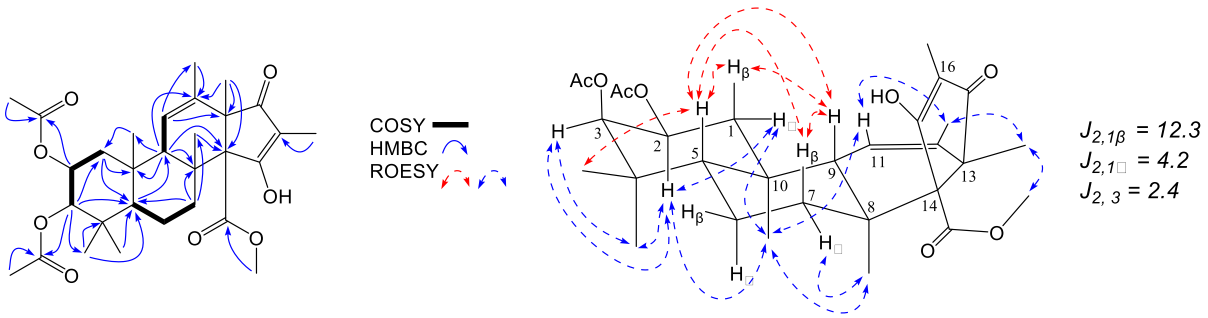

Figure S9. Key 2D NMR (600 MHz, MeOH-*d*$_4$) correlations for oxandrastin A (**1**).

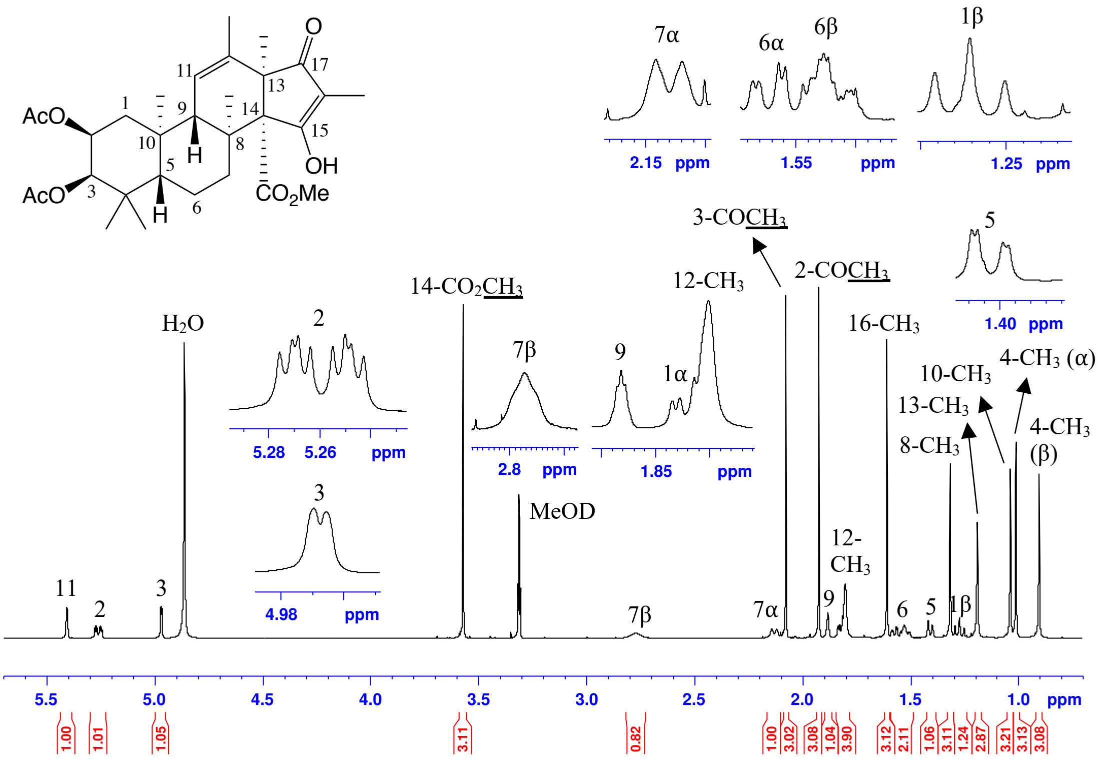

Figure S10. ^{1}H NMR (600 MHz, MeOH-d_4) spectrum for oxandrastin A (**1**).

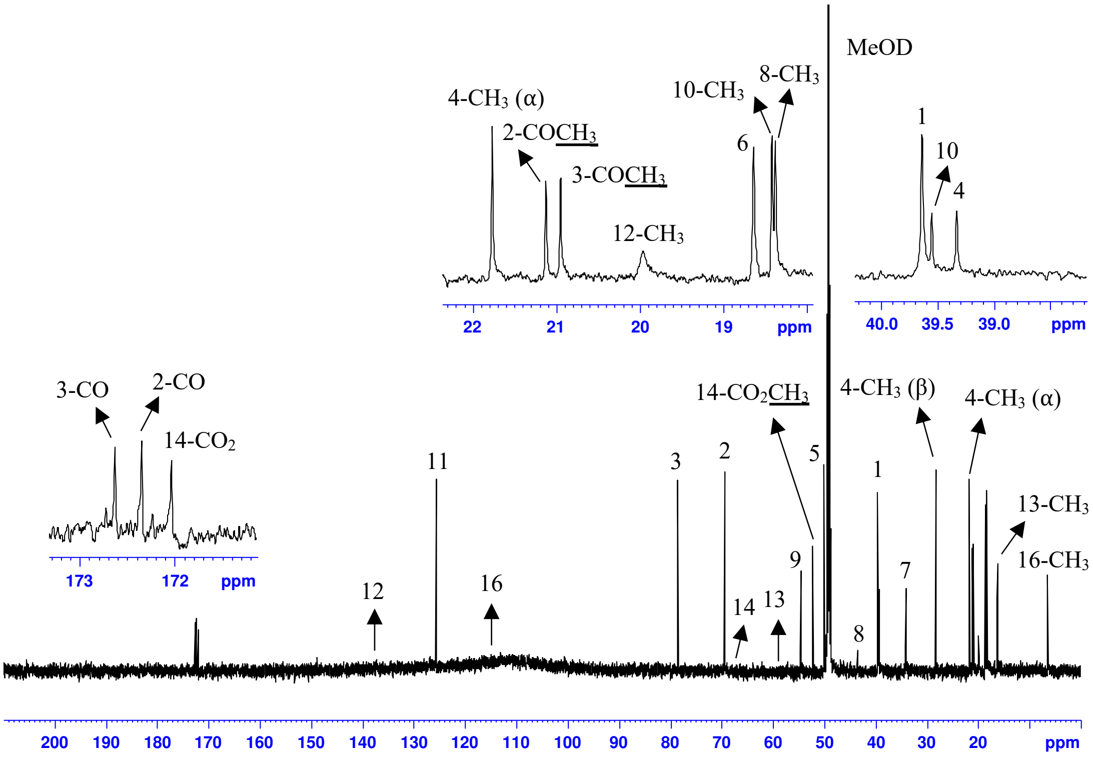

Figure S11. ^{13}C NMR (150 MHz, MeOH-d_4) spectrum for oxandrastin A (**1**).

3.1.2 Oxandrastin B (2)

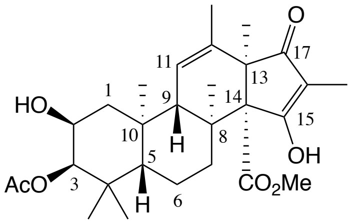

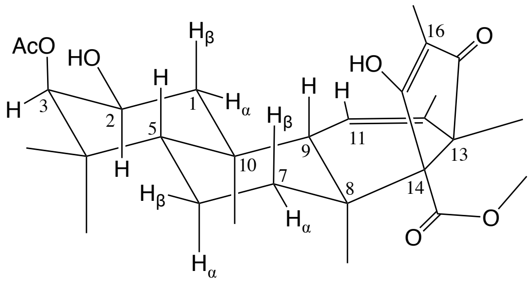

Table S3. 1D and 2D NMR (600 MHz, MeOH-d_4) data for oxandrastin B (**2**)

Position	δ_H, mult (*J* in Hz)	δ_C	COSY	^{1}H-^{13}C HMBC	ROESY
1	α 1.81, dd (*12.3, 4.1*)	42.8	1β, 2	2, 3, 5, 10, 10-CH_3	2, 10-CH_3, 11
	β 1.14, dd (*12.3, 12.1*)		1α, 2	2, 3, 9, 10, 10-CH_3	9
2	4.07, ddd (*12.1, 4.1, 2.8*)	65.6	1, 3	1, 3, 4/10	1α, 3, 4-CH_3 (α), 10-CH_3
3	4.91, d (*2.8*)	81.6	2	1, 2, 5, 3-<u>CO</u>, 4-CH_3 (α)	1α, 2, 4-CH_3 (α/β)
4	---	39.4[a]	---	---	---
5	1.33, dd (*12.1, 2.9*)	49.9	6	4/10, 7, 9, 4-CH_3 (α/β)	7β, 9, 4-CH_3 (β)
6	α 1.53, ddd (*12.8, 12.4, 3.1*)	18.6	5, 7	5, 7, 8, 4/10	
	β 1.49, m		5, 7	5, 7, 8, 4/10	
7	α 2.11, ddd (*12.8, 3.2, 3.1*)	34.1	6, 7β	5, 8, 9, 8-CH_3	8-CH_3
	β 2.75, ddd (*12.8, 12.4, 5.0*)		6, 7α	5, 8, 14, 8-CH_3	5, 9
8	---	43.5	---	---	---
9	1.85, br s	54.6	11	1, 8, 10, 11, 12, 14, 10-CH_3	1β, 5
10	---	39.4[a]	---		---
11	5.44, br s	125.9	9, 12-CH_3	8, 9, 10, 13, 12-CH_3	10-CH_3, 12-CH_3
12	---	136.9	---	---	---
13	---	58.4[b]	---	---	---
14	---	68.9[b]	---	---	---
15	---	**ND***	---	---	---
16	---	114.6	---	---	---
17	---	201.8[b]	---	---	---
3-<u>CO</u>	---	173.1	---	---	---
3-CO<u>CH$_3$</u>	2.07, s	21.2	---	3-<u>CO</u>	4-CH_3 (β)
4-CH_3 (α)	0.959, s	21.9	---	3, 4, 5, 4-CH_3 (β)	2, 3
4-CH_3 (β)	0.88, s	28.3	---	3, 4, 5, 4-CH_3 (α)	3, 5, 3-CO<u>CH$_3$</u>
8-CH_3	1.30, s	18.4	---	8, 9, 10, 14	10-CH_3
10-CH_3	0.99, s	18.5	---	1, 5, 9, 10	1α, 2, 11, 8-CH_3
12-CH_3	1.80, br s	19.9	11	11, 12, 13	11, 13-CH_3
13-CH_3	1.18, s	16.2	---	12, 13, 14, 17	12-CH_3, 14-CO_2<u>CH$_3$</u>
14-<u>CO$_2$</u>	---	172.0	---	---	---
14-CO_2<u>CH$_3$</u>	3.56, s	52.2	---	14-<u>CO$_2$</u>	13-CH_3
16-CH_3	1.59, s	6.4	---	14, 16, 17	---

(a) signals with same letter are interchangeable, (b) values determined from ^{13}C NMR and HMBC spectra, ***ND** not detected

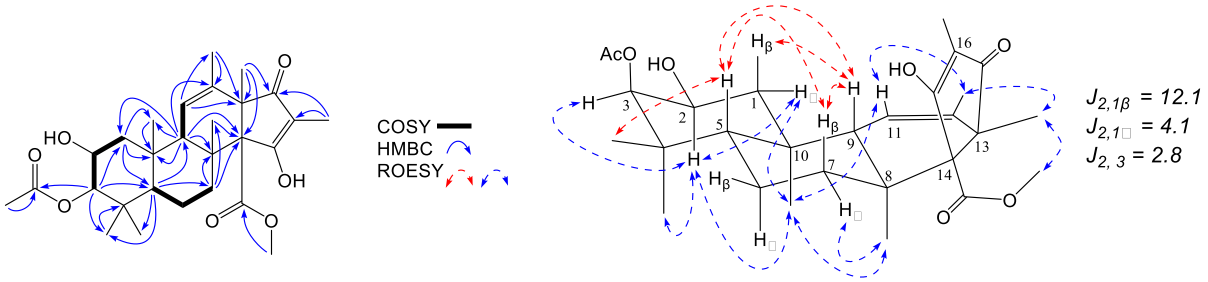

Figure S12. Key 2D NMR (600 MHz, MeOH-d_4) correlations for oxandrastin B (**2**).

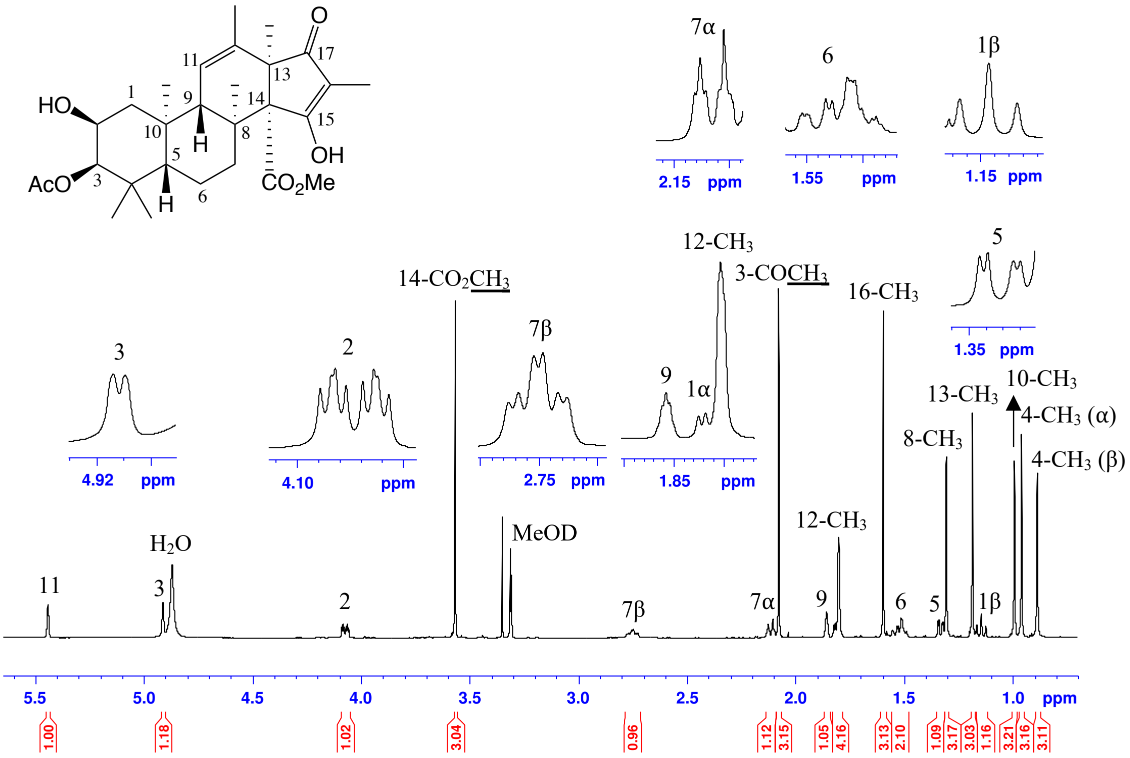

Figure S13. ^{1}H NMR (600 MHz, MeOH-d_4) spectrum for oxandrastin B (**2**).

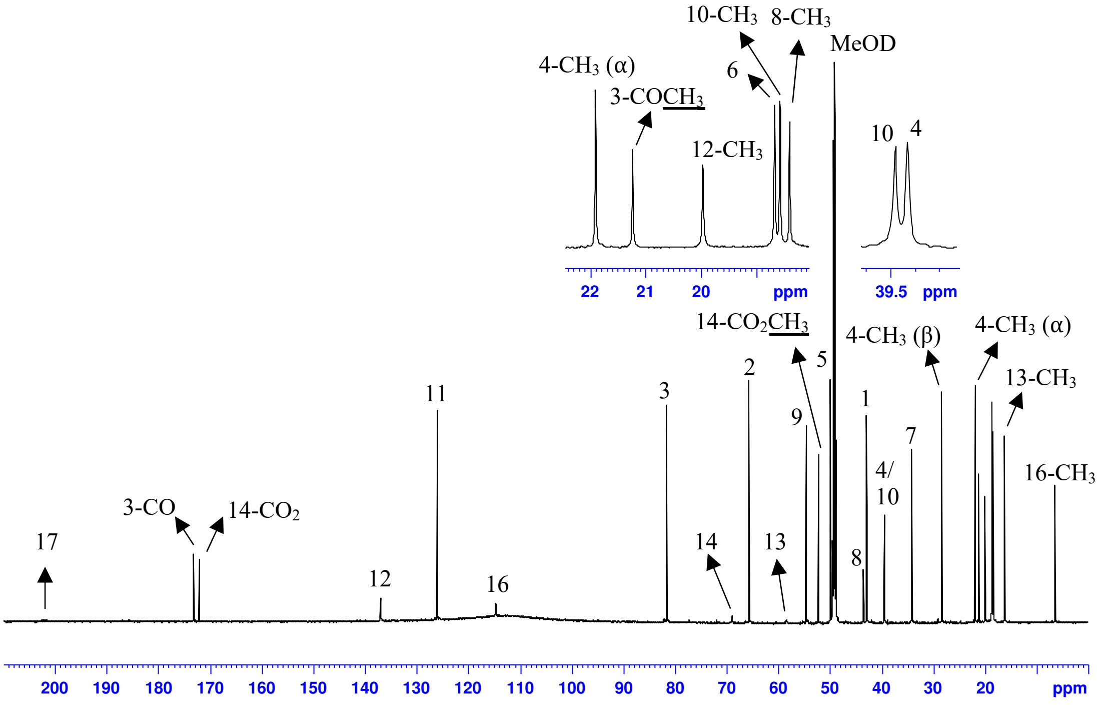

Figure S14. ^{13}C NMR (150 MHz, MeOH-d_4) spectrum for oxandrastin B (**2**).

3.1.3 Oxandrastin C (3)

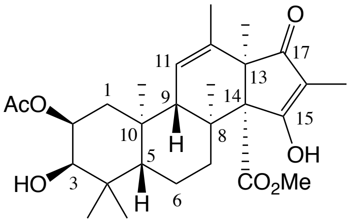

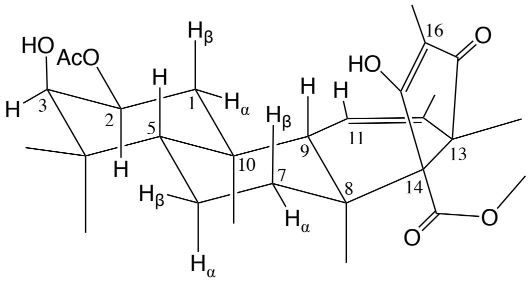

Table S4. NMR (600 MHz, MeOH-d_4) data for oxandrastin C (**3**)

Position	δ_H, mult (*J* in Hz)	δ_C	COSY	^{1}H-^{13}C HMBC	ROESY
1	α 1.72, dd (*12.0, 4.1*)	38.8	1β, 2	5, 10, 10-CH$_3$	10-CH$_3$, 11
	β 1.36, dd (*12.1, 12.0*)		1α, 2	2, 3, 9, 10, 10-CH$_3$	9
2	5.17, ddd (*12.1, 4.1, 2.7*)	71.9	1, 3	1, 3, 2-CO	3, 4-CH$_3$ (α), 10-CH$_3$
3	3.44, d (*2.7*)	77.2	2	1, 2, 5, 4-CH$_3$ (α), 4/10	2, 4-CH$_3$ (α/β)
4	---	40.0	---	---	---
5	1.45, dd (*12.1, 2.0*)	48.4	6α	1, 4/10, 7, 9, 4-CH$_3$ (α), 10-CH$_3$	7β, 9, 4-CH$_3$ (β)
6	α 1.52, ddd (*13.1, 12.8, 3.1*)	18.7	5, 6β, 7	5, 7, 8, 10	---
	β 1.46, m		5, 6α, 7	5, 7, 8, 10	---
7	α 2.09, ddd (*13.1, 3.1, 2.4*)	34.2	6, 7β	5, 6, 8, 9, 8-CH$_3$	8-CH$_3$
	β 2.76, ddd (*13.1, 12.8, 4.4*)		6, 7α	6, 8, 14, 8-CH$_3$	5, 9
8	---	43.5	---	---	---
9	1.86, br s	54.6	11	1, 8, 10, 11, 12, 14, 10-CH$_3$	1β, 5, 7β
10	---	39.4	---	---	---
11	5.39, br s	126.0	9, 12-CH$_3$	9, 10, 13, 12-CH$_3$	1α, 10-CH$_3$, 12-CH$_3$
12	---	136.7	---	---	---
13	---	58.4	---	---	---
14	---	68.8	---	---	---
15	---	**ND***	---	---	---
16	---	114.8	---	---	---
17	---	202.3[a]	---	---	---
2-<u>C</u>O	---	172.7	---	---	---
2-CO<u>CH$_3$</u>	2.03, s	21.3	---	2, 2-CO	4-CH$_3$ (β)
4-CH$_3$ (α)	0.91, s	22.0	---	3, 4, 5, 4-CH$_3$ (β)	2, 3
4-CH$_3$ (β)	0.99, s	29.1	---	3, 4, 5, 4-CH$_3$ (α)	3
8-CH$_3$	1.29, s	18.3	---	7, 8, 9, 14	7α, 10-CH$_3$
10-CH$_3$	1.00, s	18.5	---	1, 5, 9, 10	1α, 2, 11, 8-CH$_3$
12-CH$_3$	1.78, br s	19.9	11	11, 12, 13	11, 13-CH$_3$
13-CH$_3$	1.17, s	16.2	---	12, 13, 14, 17	12-CH$_3$, 14-CO$_2$<u>CH$_3$</u>
14-<u>C</u>O$_2$	---	172.1	---	---	---
14-CO$_2$<u>CH$_3$</u>	3.56, s	52.1	---	14-CO$_2$	13-CH$_3$
16-CH$_3$	1.57, s	6.4	---	14, 16, 17	---

(a) values determined from ^{13}C NMR and HMBC spectra, *ND not detected

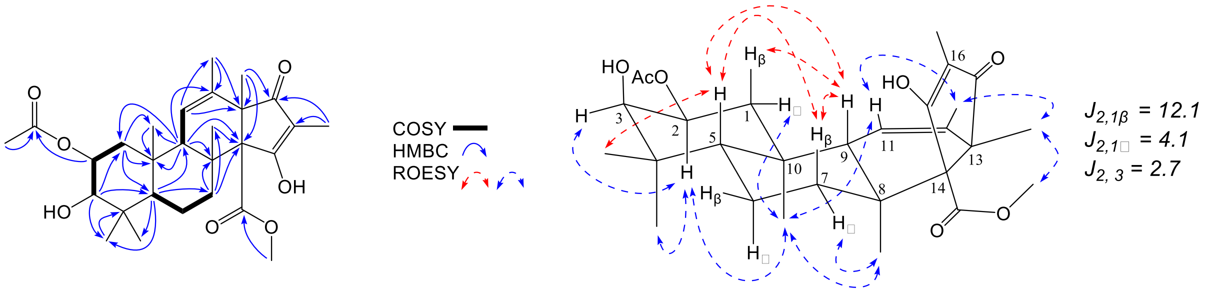

Figure S15. Key 2D NMR (600 MHz, MeOH-d_4) correlations for oxandrastin C (**3**).

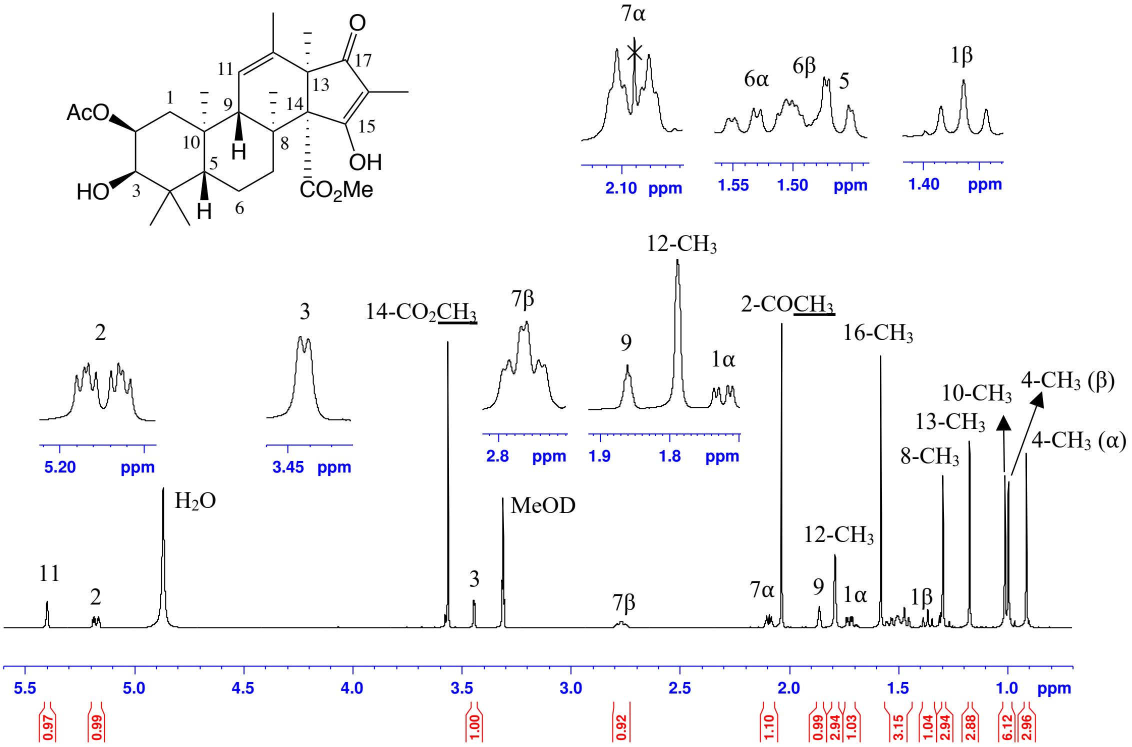

Figure S16. ^{1}H NMR (600 MHz, MeOH-d_4) spectrum for oxandrastin C (**3**).

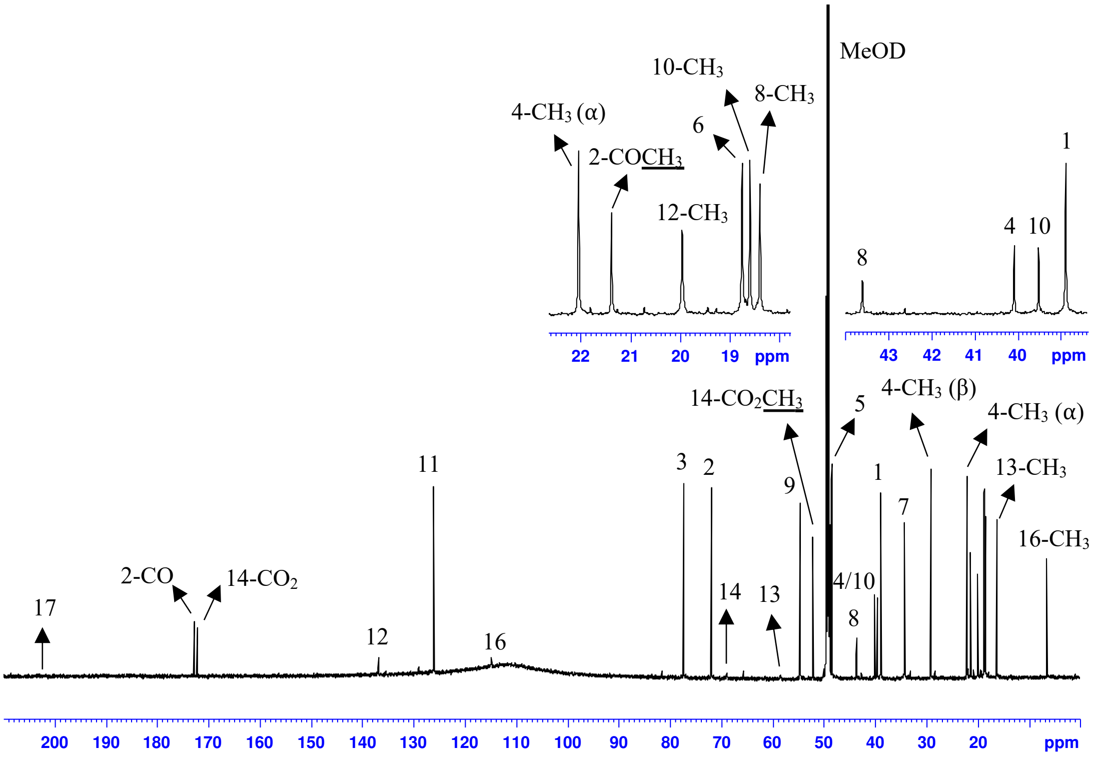

Figure S17. ^{13}C NMR (150 MHz, MeOH-d_4) spectrum for oxandrastin C (**3**).

3.1.4 Oxandrastin D (4)

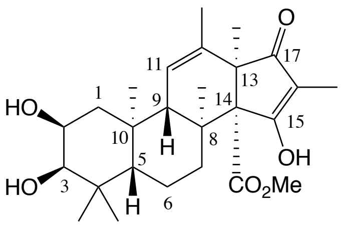

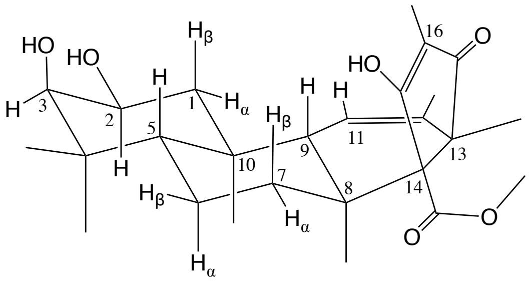

Table S5. NMR (600 MHz, MeOH-d_4) data for oxandrastin D (**4**)

Position	δ_H, mult (*J* in Hz)	δ_C	COSY	^{1}H-^{13}C HMBC
1	α 1.71, dd (*12.0, 4.1*)	42.2	1β, 2	2, 3, 5, 10
	β 1.17, dd (*12.1, 12.0*) [a]		1α, 2	2, 10-CH_3
2	3.94, ddd (*11.9, 4.3, 3.0*)	67.0	1, 3	
3	3.32, d (*3.0*) [b]	79.7	2	1, 2, 4, 5, 4-CH_3 (α)
4	---	39.6	---	---
5	1.39, dd (*11.7, 2.7*)	48.3	6	4/10, 4-CH_3 (α), 10-CH_3
6	α 1.51, (*12.8, 12.4, 3.2*)	18.8	5, 7α	5, 10
	β 1.48, m		5, 7	5, 10
7	α 2.07, ddd (*13.2, 3.0, 2.8*)		6, 7β	
	β 2.74, m	34.3	6, 7α	8-CH_3
8	---	43.6	---	---
9	1.82, br s	54.6	11	8, 10, 11, 8-CH_3/10-CH_3
10	---	39.2	---	---
11	5.44, br s	126.4	9, 12-CH_3	8, 9, 10, 12-CH_3
12	---	136.3[c]	---	---
13	---	58.8[c]	---	---
14	---	68.8[c]	---	---
15	---	**ND***	---	---
16	---	114.9[c]	---	---
17	---	**ND***	---	---
4-CH_3 (α)	0.86, s	22.1	---	3, 4, 5, 4-CH_3 (β)
4-CH_3 (β)	0.99, s	29.2	---	3, 4, 5, 4-CH_3 (α)
8-CH_3	1.28, s	18.4	---	7, 8, 9, 14
10-CH_3	0.95, s	18.7	---	1, 5, 9, 10
12-CH_3	1.79, br s	19.9	11	11, 12
13-CH_3	1.17, s[a]	16.2	---	12, 14
14-$\underline{C}O_2$	---	172.1	---	---
14-$CO_2\underline{CH_3}$	3.56, s	52.1	---	14-$\underline{C}O_2$
16-CH_3	1.57, s	6.4	---	16

(a) interchangeable signals, (b) obscured by solvent signal, (c) determined from HMBC spectrum, *ND not detected

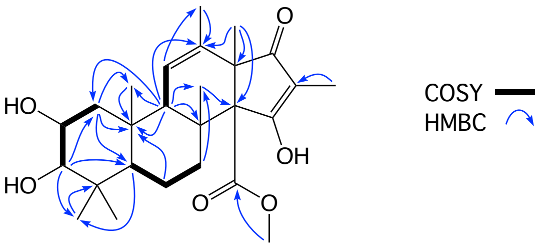

Figure S18. Key 2D NMR (600 MHz, MeOH-d_4) correlations for oxandrastin D (**4**).

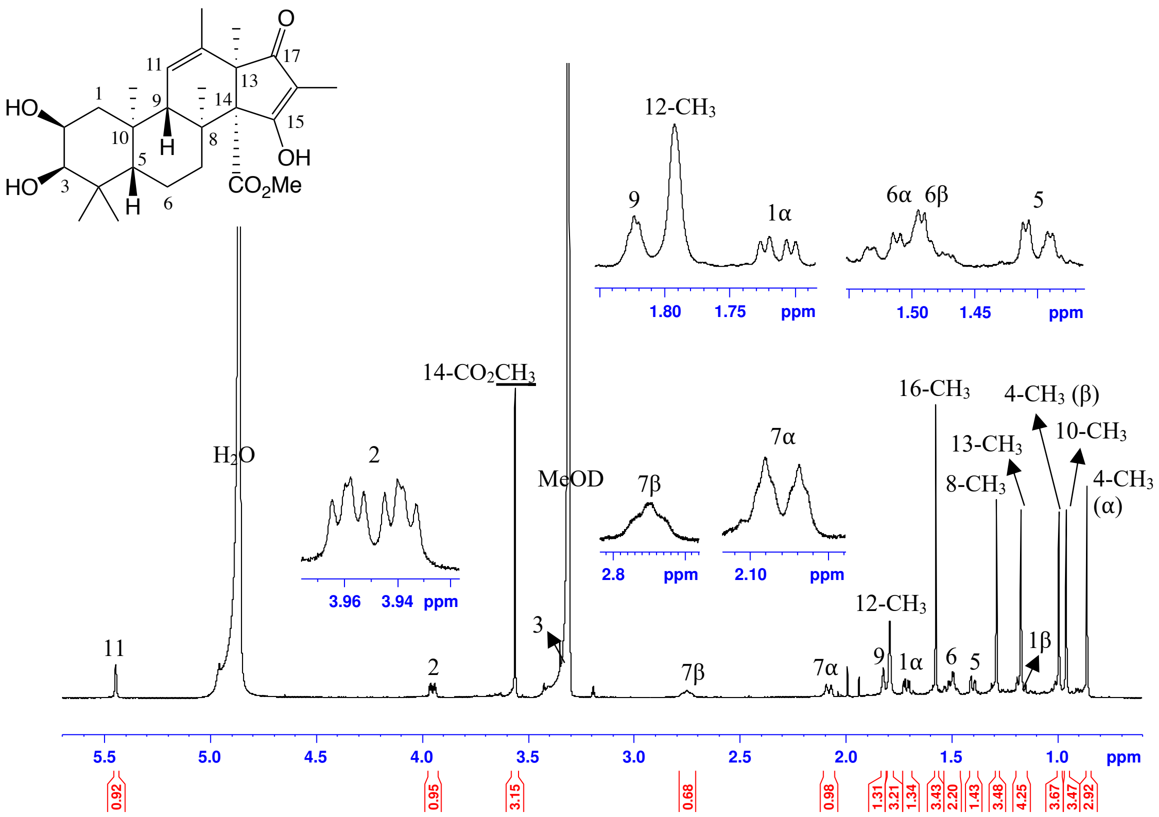

Figure S19. ^{1}H NMR (600 MHz, MeOH-d_4) spectrum for oxandrastin D (**4**).

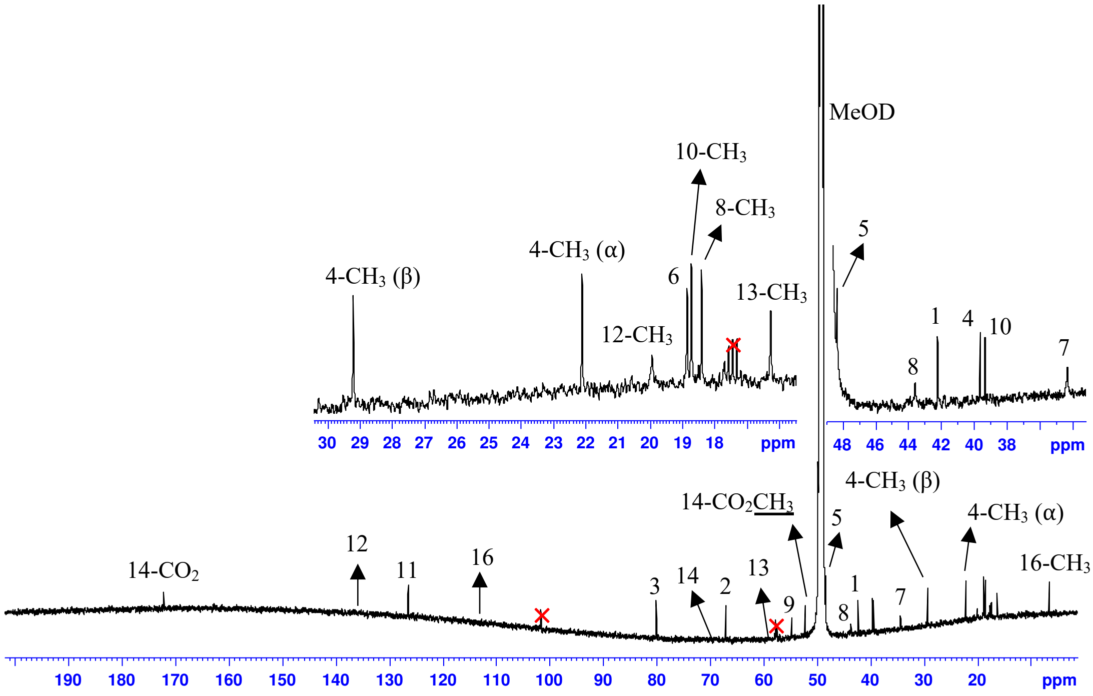

Figure S20. ^{13}**C** NMR (**150** MHz, MeOH-d_4) spectrum for oxandrastin D (**4**).

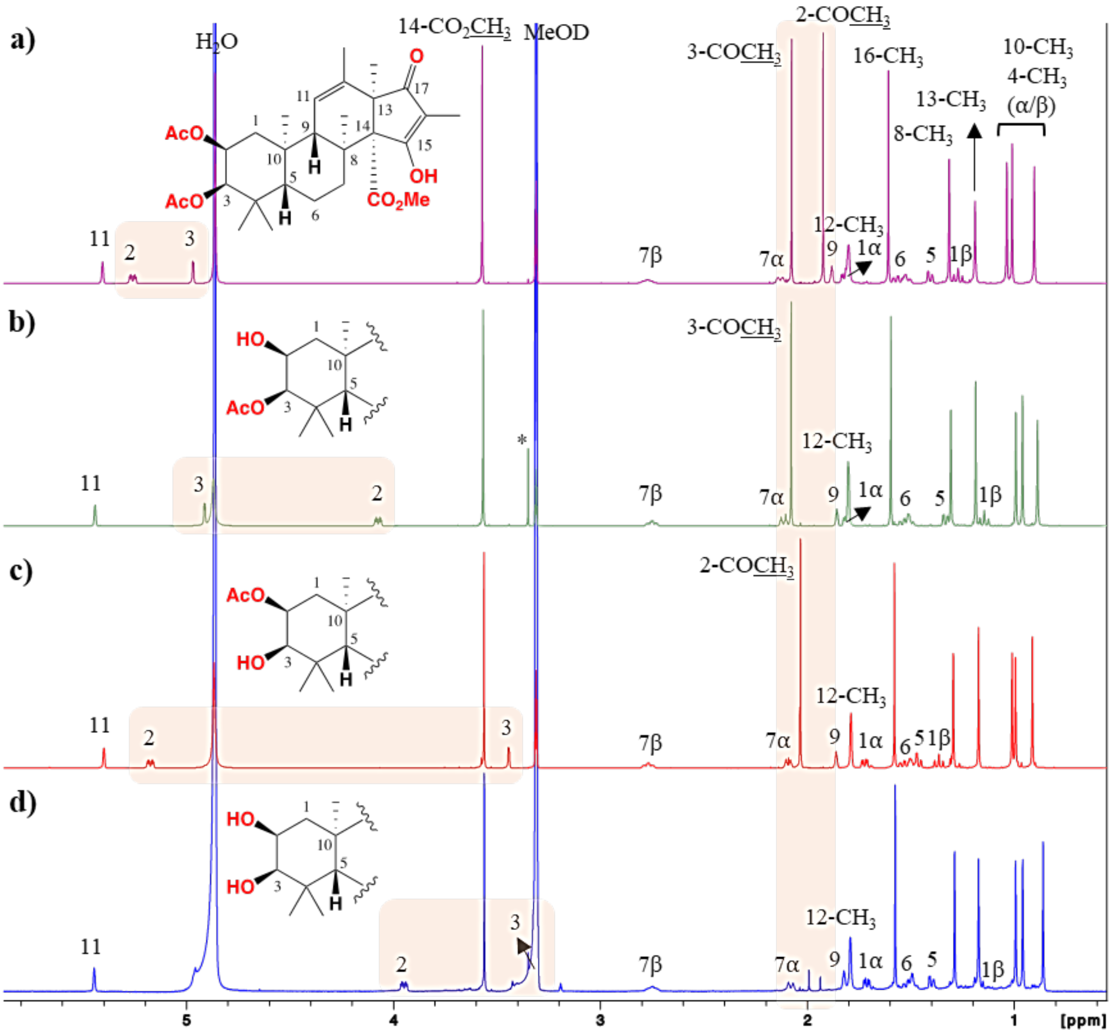

Figure S21. ^{1}H NMR (600 MHz, MeOH-d_4) spectra for oxandrastins a) A (**1**), b) B (**2**), c) C (**3**) and d) D (**4**).

3.1.5 Andrastin C (5)

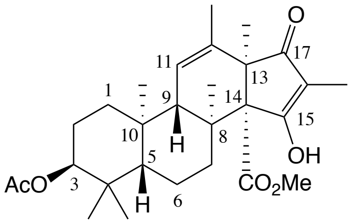

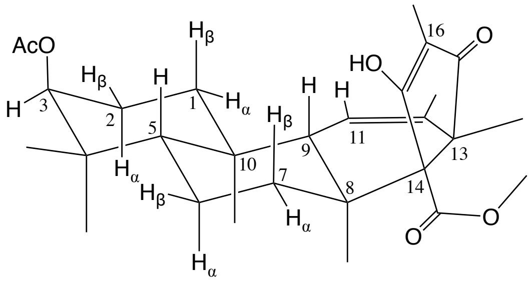

Table S6. 1D and 2D NMR (600 MHz, MeOH-d_4) data for andrastin C (**5**)

Position	**5**				reported andrastin C	
	δ_H, mult (*J* in Hz)	δ_C	COSY	^{1}H-^{13}C HMBC	δ_H, mult (*J* in Hz)	δ_C
1	α 1.57[a], m	34.5	1α, 2	overlapping	1.57, m	34.4
	β 1.12, ddd (*13.0, 12.6, 2.1*)		1β	2, 3, 9, 10, 10-CH_3	1.12, m	
2	α 1.98, dddd (*13.7, 13.7, 4.6, 2.9*)	23.6	1β, 2α, 3	1, 3, 4, 5, 10	1.96, m	23.5
	β 1.57[a], m		1α, 2β, 3	overlapping	1.59, m	
3	4.62, dd (*2.9, 2.7*)	79.7	2	1, 4, 5, 4-CH_3, 3-CO	4.63, dd (*2.4, 2.4*)	79.6
4	---	37.8	---	---	---	37.7
5	1.43, dd (*12.1, 2.4*)	50.5	6	1, 4, 7, 9, 10, 4-CH_3, 10-CH_3	1.43, m	50.3
6	α 1.55, m	18.9	5, 7α	4, 5, 7, 8, 10		18.8
	β 1.51, m		7	4, 5, 7, 8, 10	1.51, m	
7	α 2.09, ddd (*13.1, 3.1, 2.9*)	34.2	6α, 7β	5, 6, 8, 9, 8-CH_3	2.10, m	34.0
	β 2.77, ddd (*13.1, 12.6, 4.2*)		6β, 7α	5, 8, 14, 8-CH_3	2.77, m	
8	---	43.5#	---	---	---	38.1#
9	1.81, br s	54.6	11, 12-CH_3	1, 8, 10, 11, 12, 14, 8-CH_3, 10-CH_3	1.81, m	54.4
10	---	38.2#	---	---	---	43.4#
11	5.39, br s	126.3	9, 12-CH_3	8, 9, 10, 13, 12-CH_3	5.40, br s	126.3
12	---	136.5	---	---	---	136.4
13	---	58.3	---	---	---	58.1
14	---	69.0	---	---	---	68.8
15	---	**ND***	---	---	---	188.0
16	---	114.6	---	---	---	114.4
17	---	201.8[b]	---	---	---	201.7
3-<u>C</u>O	---	172.6	---	---	---	172.5
3-CO<u>CH</u>$_3$	2.03, s	21.3	---	3, 3-CO	2.03, s	21.1
4-CH_3 (α)	0.92, s	22.1	---	3, 4, 5, 4-CH_3 (β)	0.93, s	22.0
4-CH_3 (β)	0.87, s	28.3	---	3, 4, 5, 4-CH_3 (α)	0.88, s	28.2
8-CH_3	1.30, s	18.3	---	7, 8, 9, 14	1.31, s	18.1
10-CH_3	0.95, s	17.4	---	1, 5, 9, 10	0.95, s	17.3
12-CH_3	1.78, br s	19.9	9, 11	11, 12, 13	1.75, br s	19.8
13-CH_3	1.18, s	16.2	---	12, 13, 14, 17	1.18, s	16.1
14-<u>C</u>O$_2$	---	172.1	---	---	---	172.0
14-CO_2<u>CH</u>$_3$	3.56, s	52.1	---	14-CO_2	3.57, s	52.0
16-CH_3	1.59, s	6.4	---	14, 16, 17	1.60, s	6.3

(a) signals are interchangeable, (b) determined from ^{13}C NMR and HMBC spectra, *ND not detected, # carbon signals are swapped in reported data

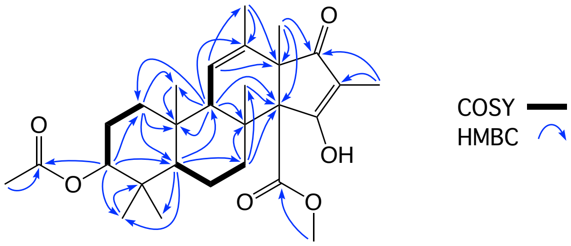

Figure S22. Key 2D NMR (600 MHz, MeOH-d_4) correlations for andrastin C (**5**).

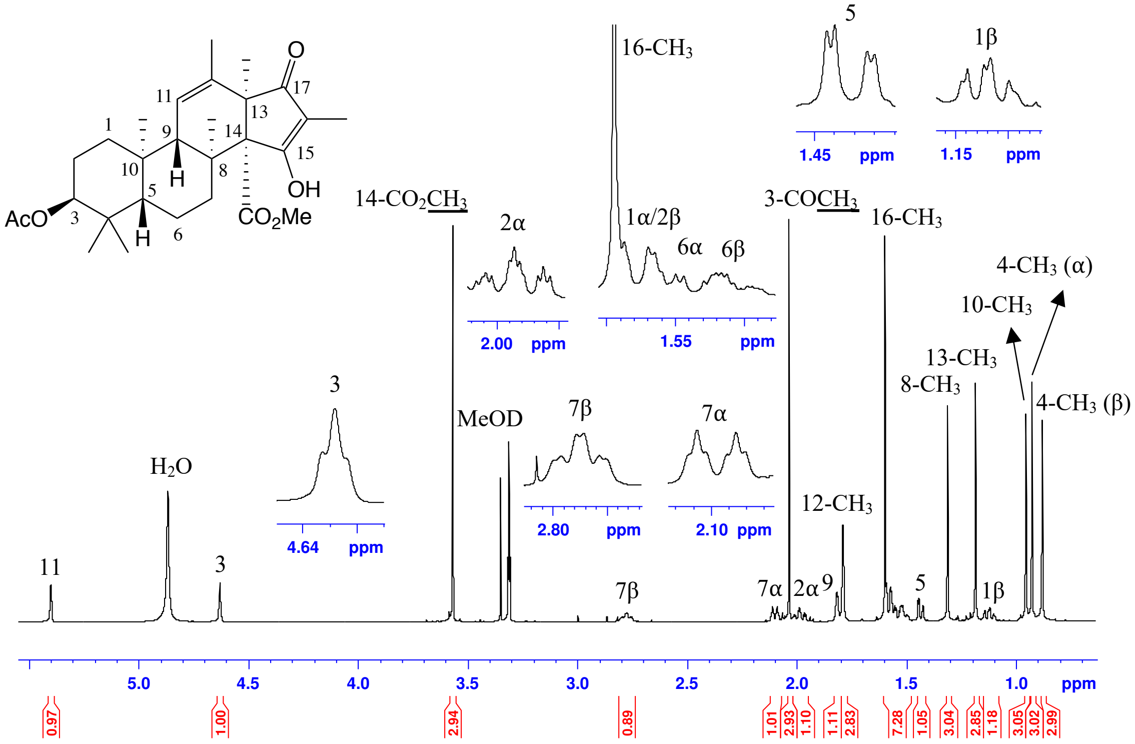

Figure S23. ^{1}H NMR (600 MHz, MeOH-d_4) spectrum for andrastin C (**5**).

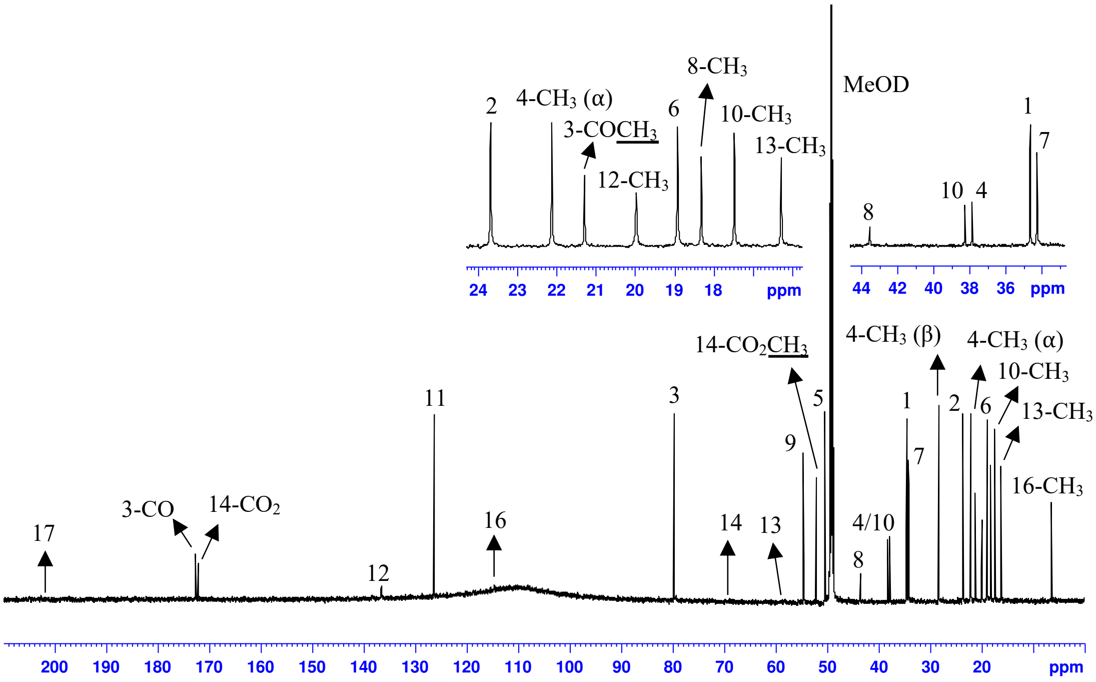

Figure S24. ^{13}C NMR (150 MHz, MeOH-d_4) spectrum for andrastin C (**5**).

3.1.6 Andrastin F (6)

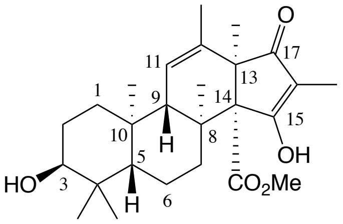

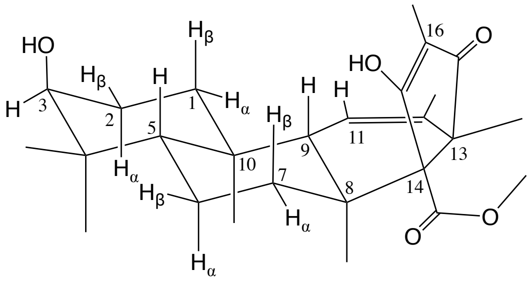

Table S7. 1D and 2D NMR (600 MHz, MeOH-d_4) data for andrastin F (**6**)

Position	**6**				reported andrastin F	
	δ_H, mult (*J* in Hz)	δ_C	COSY	^{1}H-^{13}C HMBC	δ_H, mult (*J* in Hz)	δ_C
1	α 1.47, m	34.0	1β, 2	overlapping	1.48, m	33.9
	β 1.25, ddd (*13.5, 13.3, 3.4*)		1α, 2	2, 9, 4/10, 10-CH_3	1.24, td (*13.6, 3.4*)	
2	α 1.98, dddd (*14.2, 14.2, 3.8, 2.8*)	26.1	1α, 2β, 3	1	1.98, tt (*14.2, 2.8*)	26.0
	β 1.52, m		1β, 2α, 3		1.53, m	
3	3.33, dd (*2.8, 2.8*)	76.8	2	1, 4, 5, 4-CH_3 (α)	3.34, t (*2.8*)	76.7
4	---	38.5	---	---	---	38.1
5	1.46, m	49.0	6	1, 7, 9, 6, 10, 4-CH_3, 10-CH_3	1.48, m	48.8
6	α 1.51, m	19.0	5, 7α			18.9
	β 1.46, m		7		1.48, m	
7	α 2.05, ddd (*13.2, 3.1, 2.9*)	34.3	6, 7β	5, 8, 9, 8-CH_3	2.06, dt (*13.0, 2.8*)	34.2
	β 2.81, ddd (*13.2, 12.2, 3.8*)		6, 7α	8-CH_3	2.79, td (*12.5, 5.1*)	
8	---	43.6	---	---	---	43.4
9	1.82, br s	54.6	11, 12-CH_3	8, 10, 11, 12, 14, 8-CH_3, 10-CH_3	1.81, t (*1.7*)	54.5
10	---	38.2	---	---	---	38.4
11	5.39, br s	126.5	9, 12-CH_3	8, 9, 10, 12-CH_3	5.40, br s	126.5
12	---	136.4	---	---	---	136.1
13	---	58.1	---	---	---	58.0
14	---	69.1	---	---	---	68.9
15	---	**ND***	---	---	---	187.5
16	---	114.2	---	---	---	114.3
17	---	202.2	---	---	---	202.3
4-CH_3 (α)	0.84, s	22.5	---	3, 4, 5, 4-CH_3 (β)	0.84, s	22.4
4-CH_3 (β)	0.93, s	28.9	---	3, 4, 5, 4-CH_3 (α)	0.94, s	28.8
8-CH_3	1.29, s	18.3	---	7, 8, 9, 14	1.29, s	18.2
10-CH_3	0.92, s	17.6	---	1, 5, 9, 10	0.92, s	17.5
12-CH_3	1.78, br s	20.0	9, 11	11, 12, 13	1.79, br s	19.9
13-CH_3	1.16, s	16.3	---	12, 13, 14, 17	1.17, s	16.1
14-<u>C</u>O_2	---	172.4	---	---	---	172.2
14-CO_2<u>CH_3</u>	3.55, s	52.0	---	14-CO_2	3.55, s	51.9
16-CH_3	1.56, s	6.5	---	14, 16, 17, 8, 14-CO_2	1.57, s	6.4

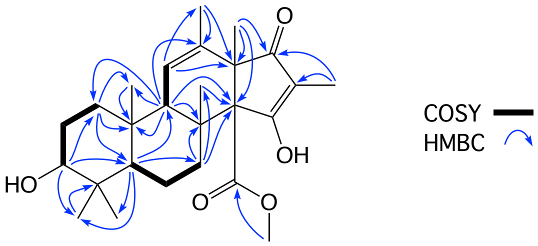

Figure S25. Key 2D NMR (600 MHz, MeOH-d_4) correlations for andrastin F (**6**).

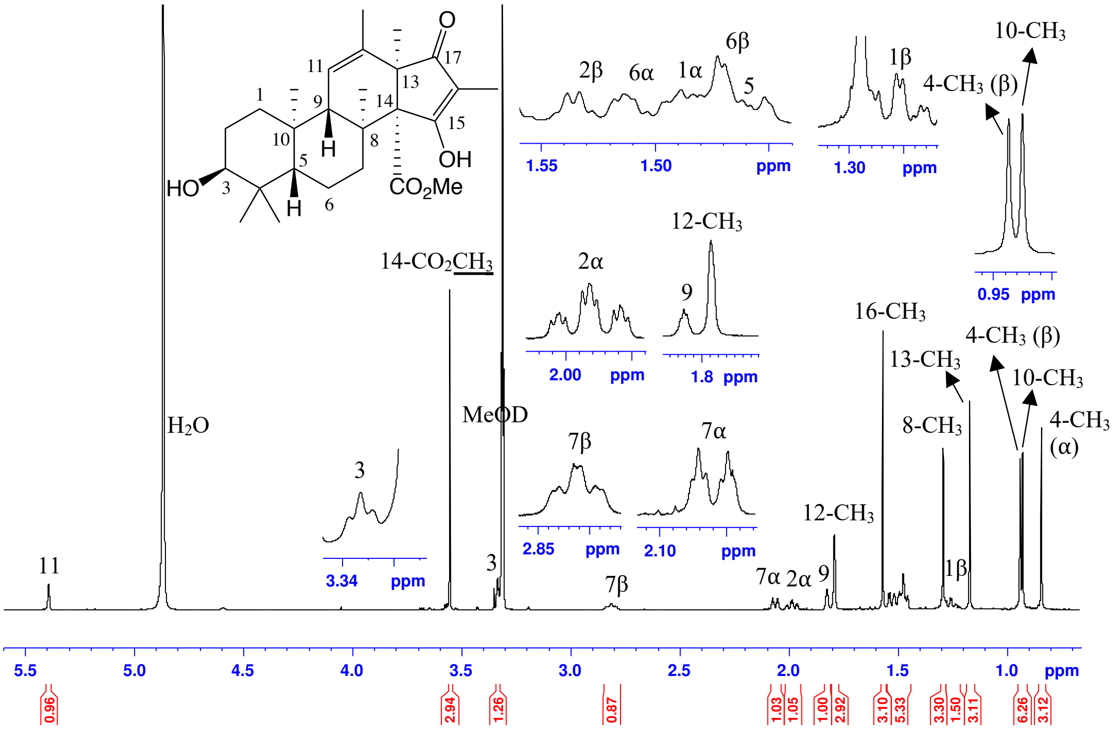

Figure S26. ^{1}H NMR (600 MHz, MeOH-d_4) spectrum for andrastin F (**6**).

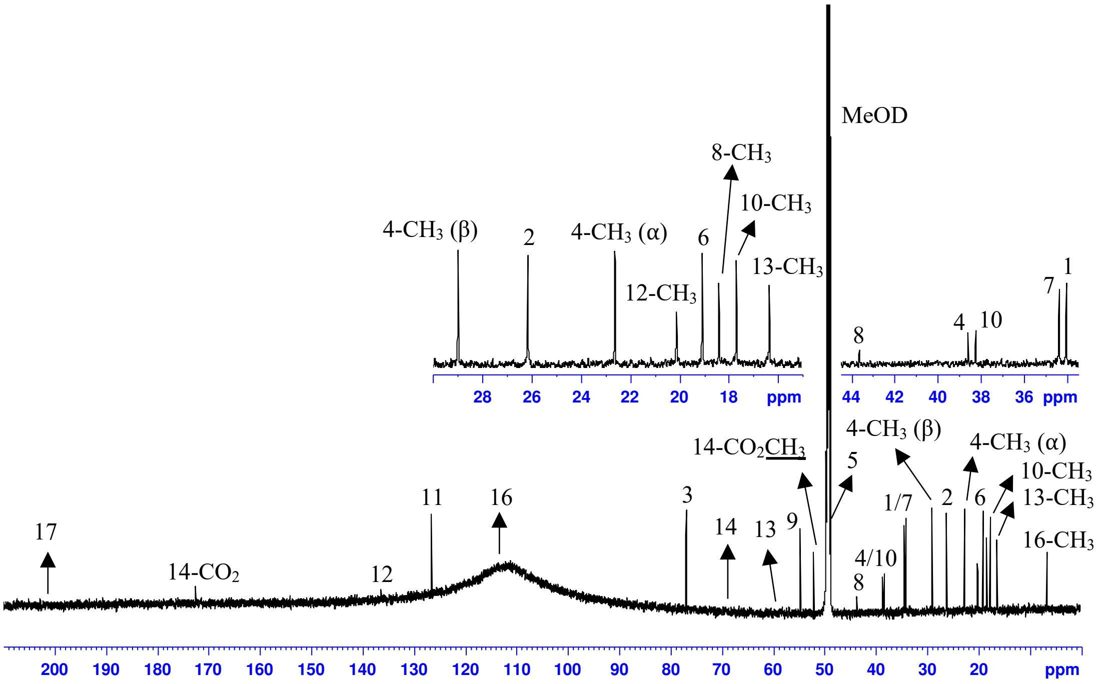

Figure S27. ^{13}C NMR (150 MHz, MeOH-d_4) spectrum for andrastin F (**6**).

3.1.7 Isoaustalide F (7)

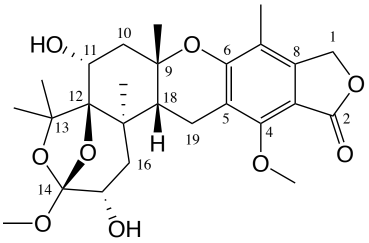

Table S8. 1D and 2D NMR (600 MHz, MeOH-d_4) data for isoaustalide F (**7**)

Position	δ_H, mult (*J* in Hz)	δ_C	COSY	^{1}H-^{13}C HMBC	ROESY
1	5.21, br s	69.9	7-CH3	3, 4, 5, 7, 8, 9	8-CH3
2	---	171.9	---	---	---
3	---	108.4	---	---	---
4	---	156.7	---	---	---
5	---	117.5	---	---	---
6	---	160.0	---	---	---
7	---	116.2	---	---	---
8	---	147.6	---	---	---
9	---	77.6	---	---	---
10	a 2.33, dd (*15.7, 2.0*)	43.4	10b, 11	9, 11, 12, 18, 9-CH3	11, 9-CH3
	b 2.16, dd (*15.7, 4.3*)		10a, 11	6, 11, 12, 9-CH3	11, 9-CH3
11	4.16, dd (*3.9, 2.0*)	69.7	10	9, 10, 12/13, 17	10a, 10b, 13-CH3(a)
12	---	87.5	---	---	---
13	---	87.0	---	---	---
14	---	120.1	---	---	---
15	3.71, dd (*10.5, 5.9*)	68.8	16	14, 16	18, 19a, 19b
16	a 2.18, dd (*13.9, 5.9*)	40.5	16b, 15	12, 14, 15, 17, 18, 17-CH3	16b, 19, 17-CH3
	b 1.68, dd (*13.9, 10.5*)		16a, 15	9, 14, 15, 17, 18, 17-CH3	16a, 17-CH3
17	---	44.6	---	---	---
18	2.40, d (*8.1*)	39.4	19b, 17-CH3	5, 9, 16, 17, 19, 9-CH3/17-CH3	10b, 15, 16a, 19a, 9-CH3
19	a 3.01, d (*18.6*)	19.2	19b	4, 5, 6, 9, 17, 18	15, 16a, 18, 9-CH3, 17-CH3, 4-OCH3
	b 2.94, dd (*18.6, 8.1*)		19a, 18	4, 5, 6, 17, 18	15, 16a, 9-CH3, 17-CH3, 4-OCH3
7-CH3	2.07, s	10.8	1	6, 7, 8	1
9-CH3	1.23, s	28.3	---	9, 10, 18	10a, 10b, 18, 19
13-CH3(a)	1.52, s	29.3	---	11, 12, 13, 13-CH3(b)	11, 14-OCH3
13-CH3(b)	1.63, s	26.7	---	12, 13, 13-CH3(a)	17-CH3
17-CH3	1.03, s	18.8	---	12, 15, 16, 17, 18	16a, 19, 13-CH3(b)
4-OCH3	4.06, s	62.4	---	4	---
14-OCH3	3.41, s	49.3[a]	---	14	---

(a) obscured by solvent signal

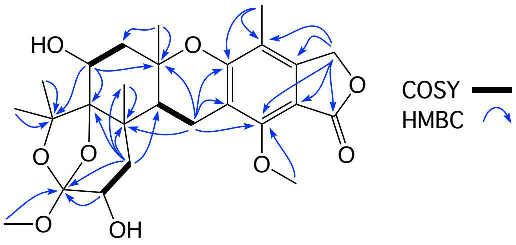

Figure S 28. Key 2D NMR (600 MHz, MeOH-d_4) correlations for isoaustalide F (**7**).

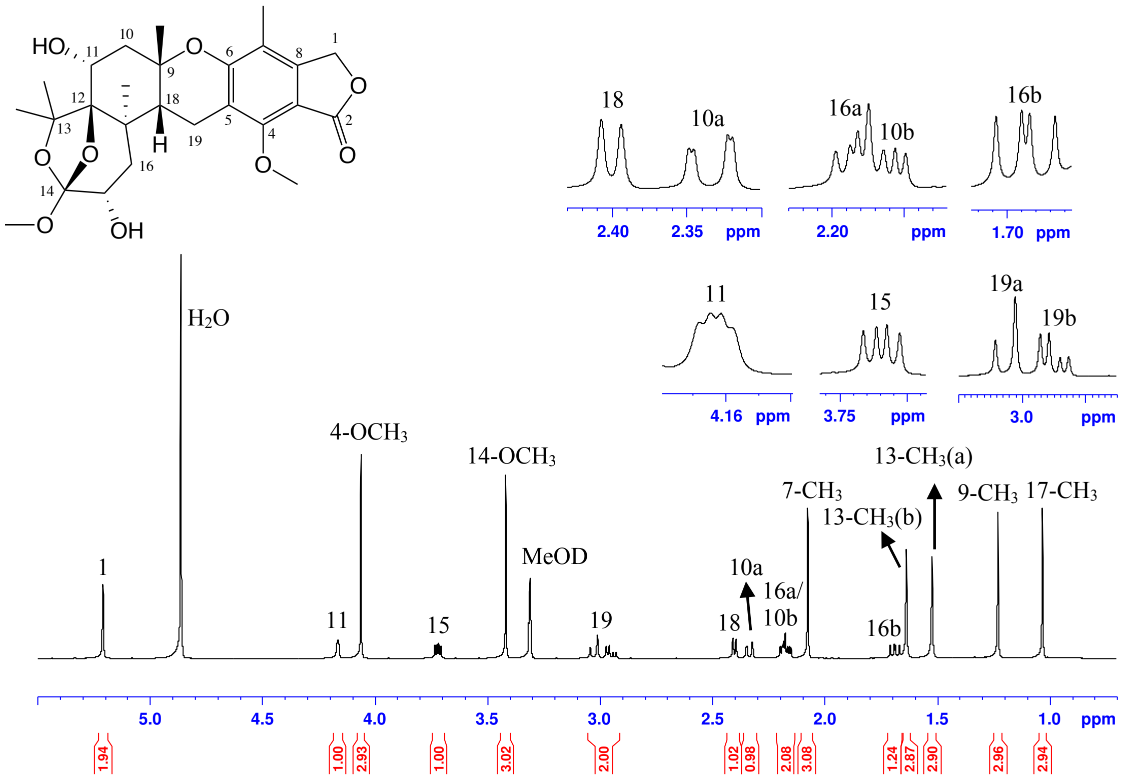

Figure S29. ^{1}H NMR (600 MHz, MeOH-d_4) spectrum for isoaustalide F (**7**).

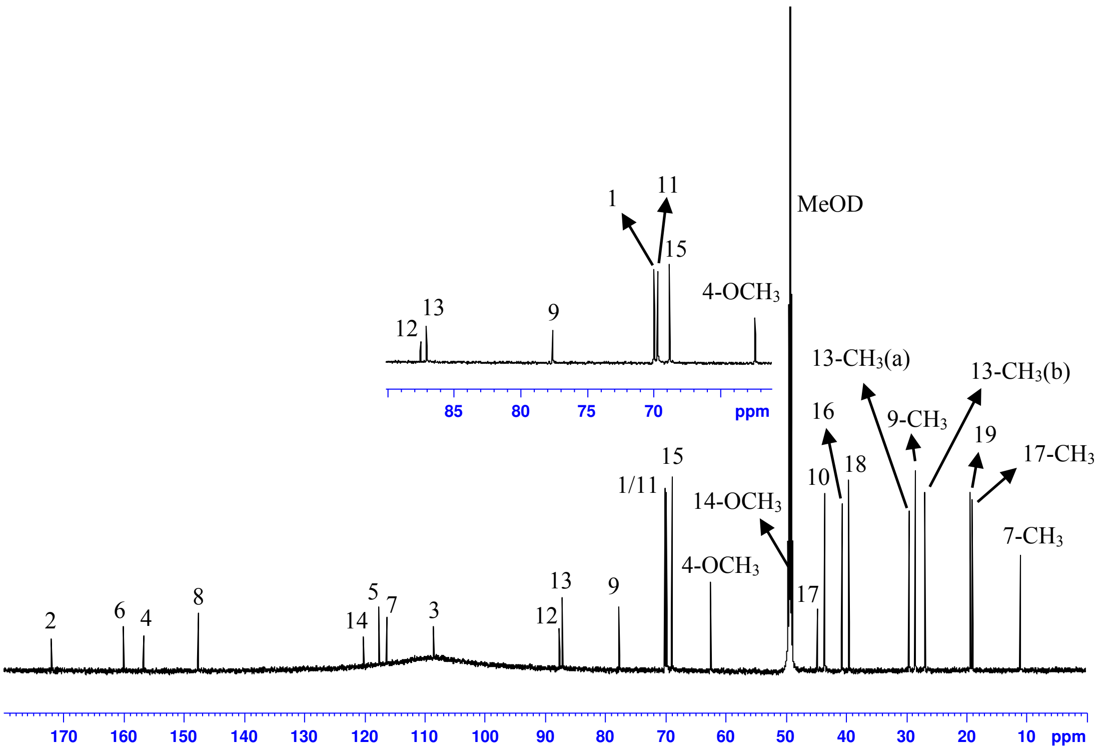

Figure S30. ^{13}C NMR (150 MHz, MeOH-d_4) spectrum for isoaustalide F (**7**).

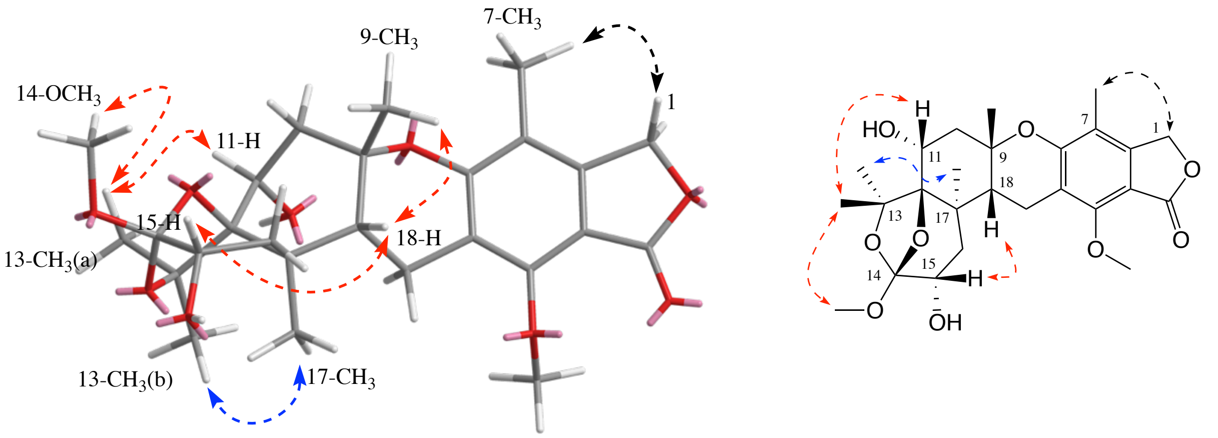

Figure S31. Energy-minimized (MM2) structure of isoaustalide F (**7**) and Key ROESY NMR (600 MHz, MeOH-d_4) correlations.

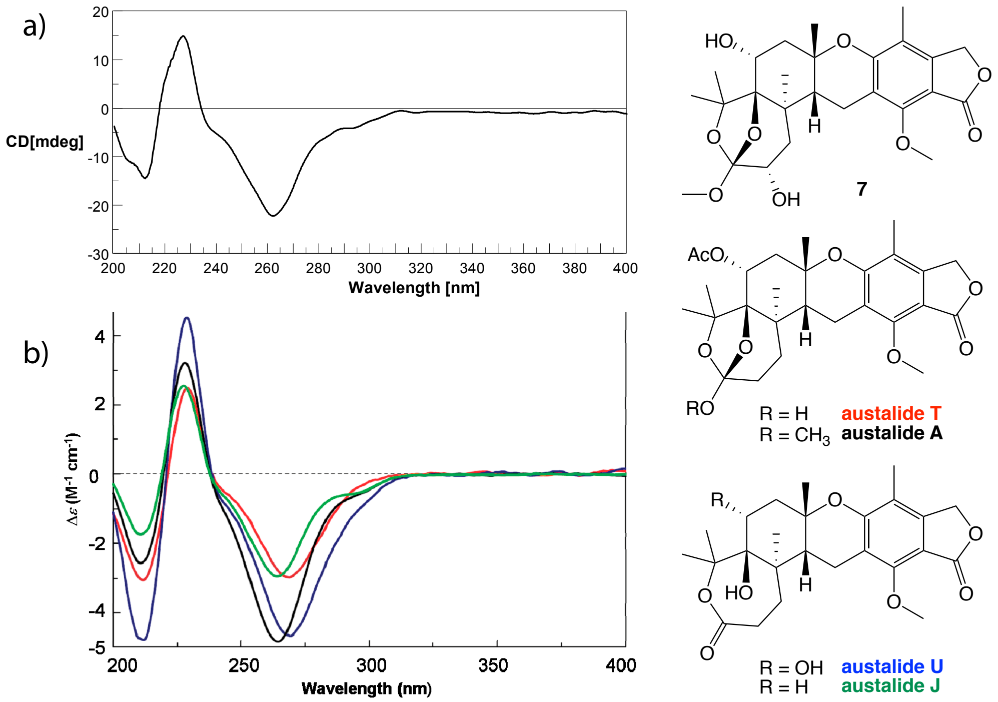

Figure S32. Experimental CD spectrum of a) isoaustalide F (**7**), 0.05% in acetonitrile compared to b) reported ECD spectra of austalides A (black), J (green), T (red) and U (blue) in acetonitrile (figure extracted from publication).[4]

4 GNPS molecular networking analysis

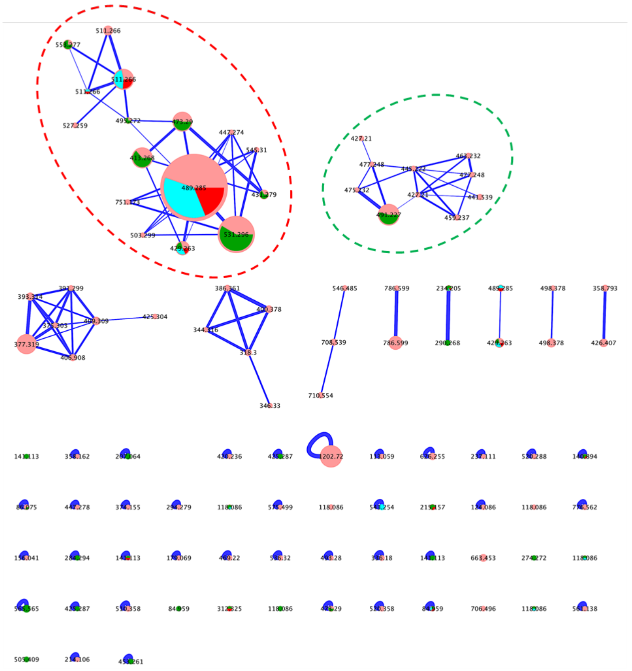

Figure S33. GNPS molecular network for CMB-MD14 rice culture. Red circle represents andrastins and oxandrastins cluster, and green circle represents austalides cluster.

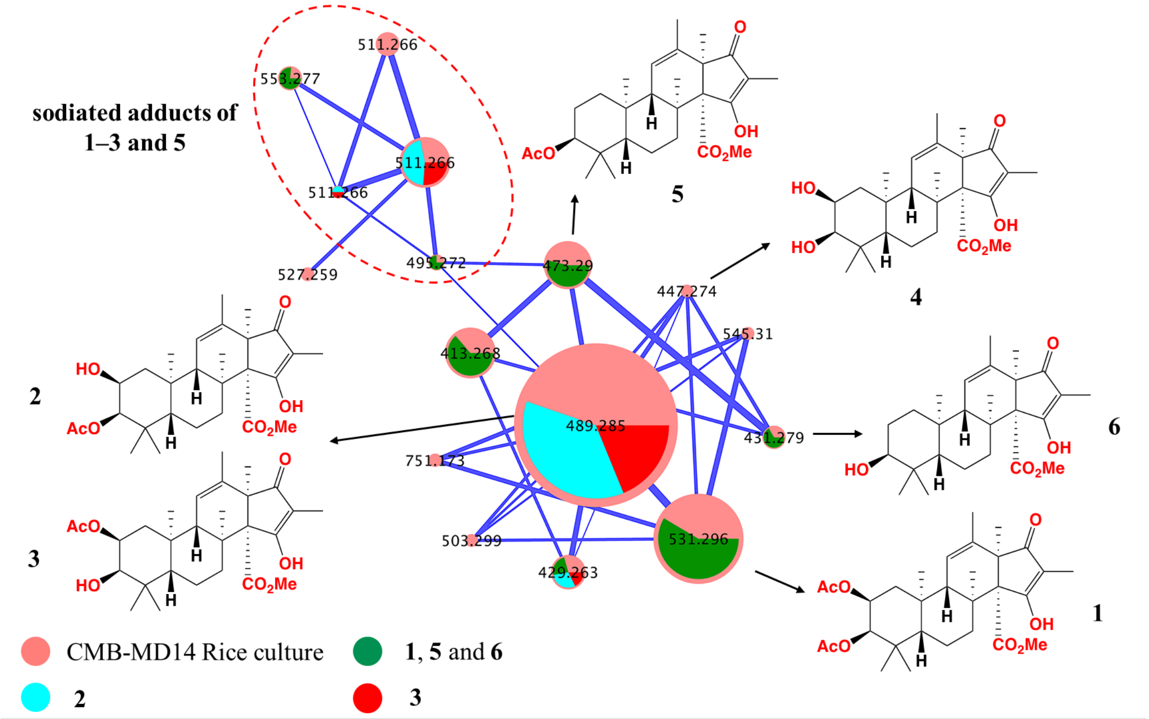

Figure S34. GNPS cluster of oxandrastins and andrastins in CMB-MD14 rice culture with the authentic compounds (**1**–**6**).

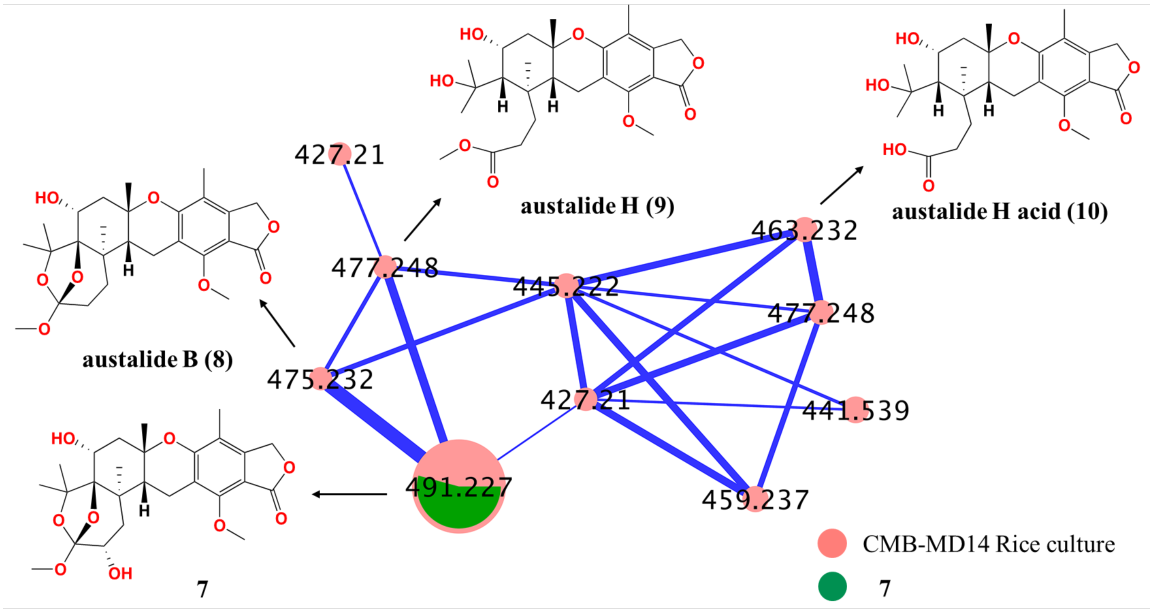

Figure S35. GNPS cluster of austalides in in CMB-MD14 rice culture with the authentic compound **7**.

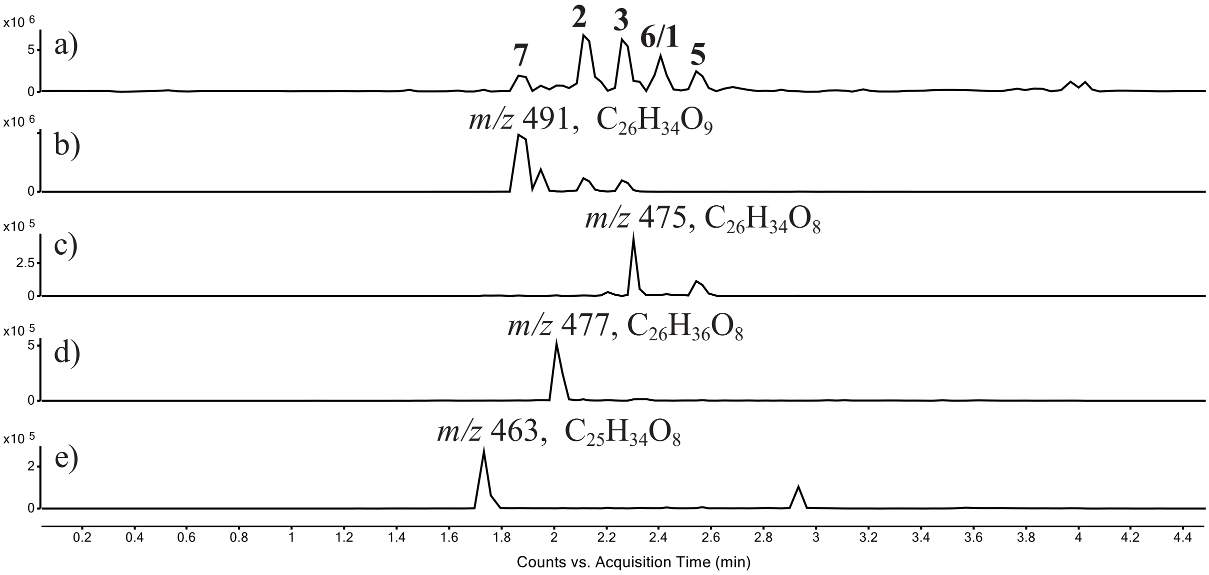

Figure S36. UPLC-QTOF analysis of austalides in CMB-MD14 rice crude extract. a) TIC of the crude extract; b) SIE for *m/z* $[M+H]^+$ 491 (isoaustalide F (**7**)); c) SIE for *m/z* $[M+H]^+$ 475 (austalide B (**8**), $C_{26}H_{34}O_8$, Δ mDa + 0.16); d) SIE for *m/z* $[M+H]^+$ 477 (austalide H (**9**), $C_{26}H_{36}O_8$, Δ mDa + 0.34); e) SIE for *m/z* $[M+H]^+$ 463 (austalide H acid (**10**), $C_{25}H_{34}O_8$, Δ mDa + 0.56).

5 Biological screening

5.1 Anti-microbial

Antibacterial assay. The bacterium to be tested was streaked onto a tryptic soy agar plate and was incubated at 37 °C for 24 h. One colony was then transferred to fresh tryptic soy broth (15 mL) and the cell density was adjusted to 7 x 10^5 CFU/mL. The compounds to be tested were dissolved in DMSO and diluted with H_2O to give 600 µM stock solution (20% DMSO), which was serially diluted with 20% DMSO to give concentrations from 600 µM to 0.2 µM in 20% DMSO. An aliquot (10 µL) of each dilution was transferred to a 96-well microtiter plate and freshly prepared microbial broth (190 µL) was added to each well to give final concentrations of 30 – 0.01 µM in 1% DMSO. The plates were incubated at 37 °C for 24 h and the optical density of each well was measured spectrophotometrically at 600 nm using POLARstar Omega plate (BMG LABTECH, Offenburg, Germany). Each test compound was screened against the Gram-negative bacteria *Escherichia coli* ATCC11775 and the Gram-positive bacteria *Staphylococcus aureus* ATCC25923, *Enterococcus faecalis* ATCC29212 and the clinical isolate Vancomycin resistant Enterococci (VRE) collected from Royal Brisbane Woman Hospital (code AUS-RBWH-VRE-01). Rifampicin and ampicillin were used as a positive control (40 µg/mL in 10% DMSO), vancomycin was used as positive control for VRE while DMSO 1% served as negative control. The IC_{50} value was calculated as the concentration of the compound or antibiotic required for 50% inhibition of the bacterial cells using Prism 7.0 (GraphPad Software Inc., La Jolla, CA).

Antifungal assay. The fungus *Candida albicans* ATCC10231 was streaked onto a Sabouraud agar plate and was incubated at 37 °C for 48 h. One colony was then transferred to fresh Sabouraud broth (15 mL) and the cell density adjusted to 10^4-10^5 CFU/mL. Test compounds were dissolved in DMSO and diluted with H_2O to give a 600 µM stock solution (20% DMSO), which was serially diluted with 20% DMSO to give concentrations from 600 µM to 0.2 µM in 20% DMSO. An aliquot (10 µL) of each dilution was transferred to a 96-well microtiter plate and freshly prepared fungal broth (190 µL) was added to each well to give final concentrations of 30 – 0.01 µM in 1% DMSO. The plates were incubated at 37 °C for 24 h and the optical density of each well was measured spectrophotometrically at 600 nm using POLARstar Omega plate (BMG LABTECH, Offenburg, Germany). Amphotericin B was used as a positive control (30 µg/ml in 10% DMSO). Where relevant, IC_{50} value were calculated as the concentration of

the compound or antifungal drug required for 50% inhibition of the fungal cells using Prism 7.0 (GraphPad Software Inc., La Jolla, CA).

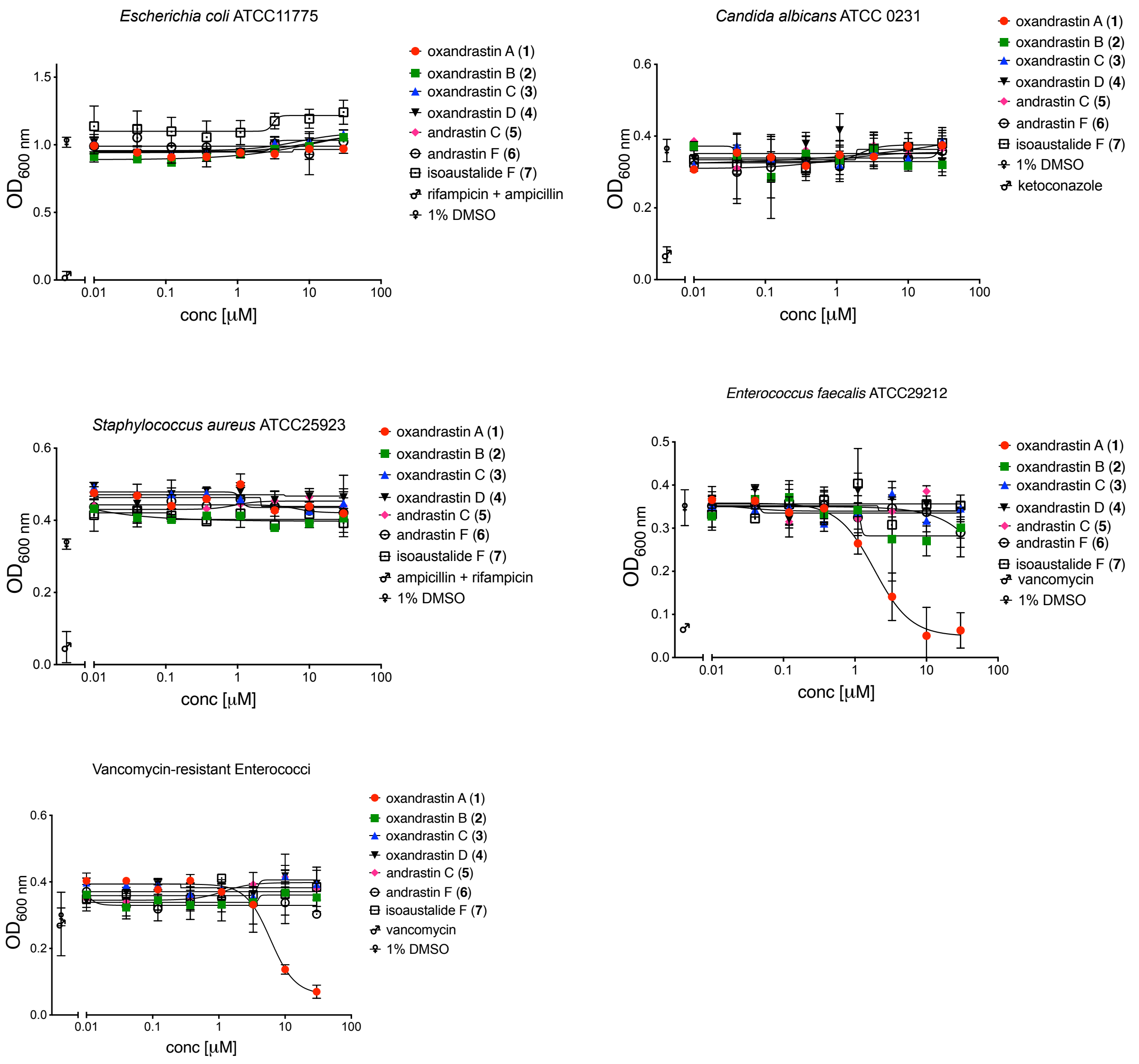

Figure S37. Anti-microbial activities of metabolites **1–7**.

5.2 Cytotoxicity assays

Adherent cell SW620 (human colorectal carcinoma) and NCI-H460 (human lung carcinoma) cells were cultured in Roswell Park Memorial Institute (RPMI) 1640 medium. All cells were cultured as adherent mono-layers in flasks supplemented with 10% foetal bovine serum, L–glutamine (2 mM), penicillin (100 unit/mL) and streptomycin (100 μg/mL), in a humidified 37 °C incubator supplied with 5% CO_2. Briefly, cells were harvested with trypsin and dispensed into 96-well microtiter assay plates at 3,000 cells/well, after which they were incubated for 18

h at 37 °C with 5% CO_2 (to allow cells to attach as adherent mono-layers). Test compounds were dissolved in 20% DMSO in PBS (v/v) and aliquots (10 μL) applied to cells over a series of final concentrations ranging from 10 nM to 30 μM. After 48 h incubation at 37 °C with 5% CO_2 an aliquot (10 μL) of 3-(4,5-dimethylthiazol-2-yl)-2,5-diphenyltetrazolium bromide (MTT) in phosphate buffered saline (PBS, 5 mg/mL) was added to each well (final concentration 0.5 mg/mL), and microtiter plates were incubated for a further 4 h at 37 °C with 5% CO_2. After final incubation, the medium was aspirated, and precipitated formazan crystals dissolved in DMSO (100 μL/well). The absorbance of each well was measured at 580 nm with a PowerWave XS Microplate Reader from Bio-Tek Instruments Inc. Where relevant, IC_{50} values were calculated using Prism 7.0, as the concentration of analyte required for 50% inhibition of cancer cell growth (compared to negative controls). Negative control was 1% aqueous DMSO, while positive control was doxorubicin (30 μM). All experiments were performed in duplicate.

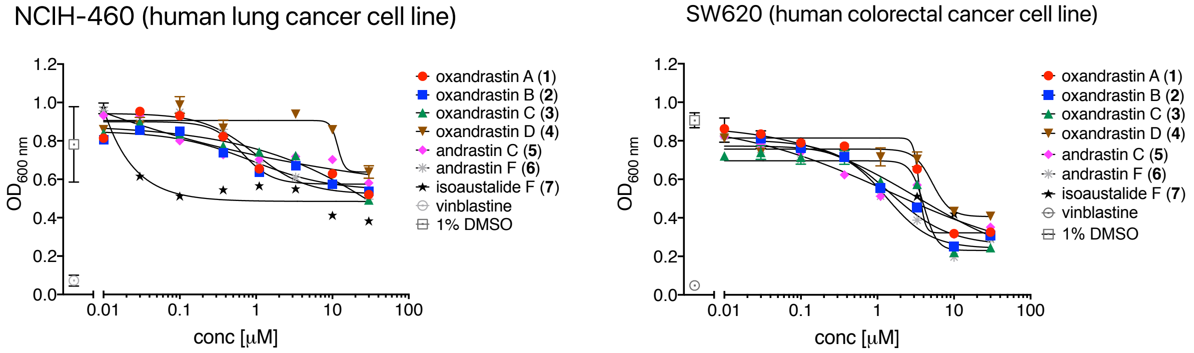

Figure S38. Cytotoxicity of metabolites **1–7**
